# Supplementary material for: Managing disease outbreaks: The importance of vector mobility and spatially heterogeneous control
Source: PLoS Comput Biol. 2020 Aug 21;16(8):e1008136. doi: 10.1371/journal.pcbi.1008136 (PMC7480881; doi:10.1371/journal.pcbi.1008136)
Supplement: S1 Appendices — This series of appendices gives mathematical expressions for the Laplacian matrices under periodic and reflecting boundary conditions (Sec. 1), gives an expression for the equilibrium vector population distribution (Sec. 2), derives mathematical expressions for the second generation matrices leading to the R0 eigenvalue problem (Sec. 3), provides detailed derivations of the R0 expressions in simplified limiting cases (Sec. 4), provides details related to the perturbation theory formalism (Sec. 5), explains the mathematical relationships between controlled model parameters and real-world control strategies’ application frequencies (Sec. 6), provides additional optimized control results for specific 20% compliance distributions (Sec. 7), and shows the relationship between total epidemic size and R0 for aerial spray and door-to-door control strategies (Sec. 8). (PDF) [file pcbi.1008136.s001.pdf]

S1 Appendices: Mathematical details and additional results

Jeffery Demers[1*], Sharon Bewick[2], Folashade Agusto[3], Kevin A. Caillouët[4], William F. Fagan[1], Suzanne L. Robertson[5],

1 Department of Biology, University of Maryland College Park, College Park, MD, United States
2 Department of Biological Sciences, Clemson University, Clemson, SC, United States
3 Department of Ecology and Evolutionary Biology, University of Kansas, Lawrence, KS, United States
4 St. Tammany Parish Mosquito Abatement District, Slidell, LA, United States
5 Department of Mathematics and Applied Mathematics, Virginia Commonwealth University, Richmond, VA, United States

* jdemers@umd.edu

1 Periodic and reflecting boundary condition Laplacian matrices

Let $0_{\mathcal{N}}$ and $1_{\mathcal{N}}$ denote the $\mathcal{N} \times \mathcal{N}$ zero and identity matrices, respectively, and let $\mathcal{L}^{pe}$ and $\mathcal{L}^{re}$ denote the periodic and reflecting boundary condition matrices, respectively. Writing $\mathcal{L}^{pe}$ in block matrix form, we have:

$$\mathcal{L}^{pe} = \begin{pmatrix} T_{\mathcal{N}} & -1_{\mathcal{N}} & 0_{\mathcal{N}} & \dots & 0_{\mathcal{N}} & 0_{\mathcal{N}} & -1_{\mathcal{N}} \\ -1_{\mathcal{N}} & T_{\mathcal{N}} & -1_{\mathcal{N}} & \dots & 0_{\mathcal{N}} & 0_{\mathcal{N}} & 0_{\mathcal{N}} \\ 0_{\mathcal{N}} & -1_{\mathcal{N}} & T_{\mathcal{N}} & \dots & 0_{\mathcal{N}} & 0_{\mathcal{N}} & 0_{\mathcal{N}} \\ \vdots & \vdots & \vdots & \ddots & \vdots & \vdots & \vdots \\ 0_{\mathcal{N}} & 0_{\mathcal{N}} & 0_{\mathcal{N}} & \dots & T_{\mathcal{N}} & -1_{\mathcal{N}} & 0_{\mathcal{N}} \\ 0_{\mathcal{N}} & 0_{\mathcal{N}} & 0_{\mathcal{N}} & \dots & -1_{\mathcal{N}} & T_{\mathcal{N}} & -1_{\mathcal{N}} \\ -1_{\mathcal{N}} & 0_{\mathcal{N}} & 0_{\mathcal{N}} & \dots & 0_{\mathcal{N}} & -1_{\mathcal{N}} & T_{\mathcal{N}} \end{pmatrix}, \tag{1}$$

where $T_{\mathcal{N}}$ denotes the following $\mathcal{N} \times \mathcal{N}$ matrix:

$$T_{\mathcal{N}} = \begin{pmatrix} 4 & -1 & 0 & \dots & 0 & 0 & -1 \\ -1 & 4 & -1 & \dots & 0 & 0 & 0 \\ 0 & -1 & 4 & \dots & 0 & 0 & 0 \\ \vdots & \vdots & \vdots & \ddots & \vdots & \vdots & \vdots \\ 0 & 0 & 0 & \dots & 4 & -1 & 0 \\ 0 & 0 & 0 & \dots & -1 & 4 & -1 \\ -1 & 0 & 0 & \dots & 0 & -1 & 4 \end{pmatrix}. \tag{2}$$

Similarly, for reflecting boundary conditions, we have

$$\mathcal{L}^{re} = \begin{pmatrix} \widetilde{\widetilde{T_{\mathcal{N}}}} & -1_{\mathcal{N}} & 0_{\mathcal{N}} & \dots & 0_{\mathcal{N}} & 0_{\mathcal{N}} & 0_{\mathcal{N}} \\ -1_{\mathcal{N}} & \widetilde{T_{\mathcal{N}}} & -1_{\mathcal{N}} & \dots & 0_{\mathcal{N}} & 0_{\mathcal{N}} & 0_{\mathcal{N}} \\ 0_{\mathcal{N}} & -1_{\mathcal{N}} & \widetilde{T_{\mathcal{N}}} & \dots & 0_{\mathcal{N}} & 0_{\mathcal{N}} & 0_{\mathcal{N}} \\ \vdots & \vdots & \vdots & \ddots & \vdots & \vdots & \vdots \\ 0_{\mathcal{N}} & 0_{\mathcal{N}} & 0_{\mathcal{N}} & \dots & \widetilde{T_{\mathcal{N}}} & -1_{\mathcal{N}} & 0_{\mathcal{N}} \\ 0_{\mathcal{N}} & 0_{\mathcal{N}} & 0_{\mathcal{N}} & \dots & -1_{\mathcal{N}} & \widetilde{T_{\mathcal{N}}} & -1_{\mathcal{N}} \\ 0_{\mathcal{N}} & 0_{\mathcal{N}} & 0_{\mathcal{N}} & \dots & 0_{\mathcal{N}} & -1_{\mathcal{N}} & \widetilde{\widetilde{T_{\mathcal{N}}}} \end{pmatrix}, \tag{3}$$

where $\widetilde{T_{\mathcal{N}}}$ and $\widetilde{\widetilde{T_{\mathcal{N}}}}$ are denote the following $\mathcal{N} \times \mathcal{N}$ matrices:

$$\widetilde{T_{\mathcal{N}}} = \begin{pmatrix} 3 & -1 & 0 & \dots & 0 & 0 & 0 \\ -1 & 4 & -1 & \dots & 0 & 0 & 0 \\ 0 & -1 & 4 & \dots & 0 & 0 & 0 \\ \vdots & \vdots & \vdots & \ddots & \vdots & \vdots & \vdots \\ 0 & 0 & 0 & \dots & 4 & -1 & 0 \\ 0 & 0 & 0 & \dots & -1 & 4 & -1 \\ 0 & 0 & 0 & \dots & 0 & -1 & 3 \end{pmatrix}, \tag{4}$$

and

$$\widetilde{\widetilde{T_{\mathcal{N}}}} = \begin{pmatrix} 2 & -1 & 0 & \dots & 0 & 0 & 0 \\ -1 & 4 & -1 & \dots & 0 & 0 & 0 \\ 0 & -1 & 4 & \dots & 0 & 0 & 0 \\ \vdots & \vdots & \vdots & \ddots & \vdots & \vdots & \vdots \\ 0 & 0 & 0 & \dots & 4 & -1 & 0 \\ 0 & 0 & 0 & \dots & -1 & 4 & -1 \\ 0 & 0 & 0 & \dots & 0 & -1 & 2 \end{pmatrix}. \tag{5}$$

2 Equilibrium vector distribution

In order to define the next generation and second generation matrices, as well as solve the corresponding eigenvalue problems, we require the equilibrium solution to the mosquito population dynamics defined in Methods I of the main text, which can be written in vector-matrix form as:

$$\dot{\mathbf{N}}^v = \mathbf{\Lambda} - (\mu_{\mathcal{N}^2} + \omega\mathcal{L})\mathbf{N}^v. \tag{6}$$

Here, $\mathbf{N}^v$ and $\mathbf{\Lambda}$ denote $\mathcal{N}^2$- dimensional column vectors with components N_i^v and Λ_i^v, respectively, and $\mu_{\mathcal{N}^2}$ denotes the $\mathcal{N}^2 \times \mathcal{N}^2$ diagonal matrix with entries along the diagonal given by μ_i. The equilibrium mosquito population distribution, whose value in patch i we denote by N_i^{ve}, is given by the stationary solution to Eq. (6). The vector of equilibrium mosquito population values, denoted by $\mathbf{N}^{ve}$, is found to be

$$\mathbf{N}^{ve} = (\mu_{\mathcal{N}^2} + \omega\mathcal{L})^{-1}\mathbf{\Lambda}. \tag{7}$$

3 Next generation matrix, second generation matrices, and eigenvalue problem derivation

Throughout this section, let 0_k and 1_k denote the $k \times k$ zero and identity matrices respectively, for any positive integer k, and let $\mathbf{0}$ denote the $\mathcal{N}^2$ dimensional zero vector.

Following [1], we define the $4\mathcal{N}^2$ dimensional new infectious vector $\mathcal{F}$ and the $4\mathcal{N}^2$ dimensional transfer vector $\mathcal{V}$ as

$$\mathcal{F} = \begin{pmatrix} \mathbf{0} \\ \mathbf{0} \\ p^v \mathbf{E}^v \\ p^h \mathbf{E}^h \end{pmatrix}, \tag{8}$$

and

$$\mathcal{V} = \begin{pmatrix} (p^v 1_{\mathcal{N}^2} + \mu_{\mathcal{N}}^2 + \omega \mathcal{L})\mathbf{E}^v - \beta^v b \frac{1}{N^h}[\mathbf{S}^v \mathbf{I}^h] \\ p^h \mathbf{E}^h - \beta^h b \frac{1}{N^h}[\mathbf{S}^h \mathbf{I}^v] \\ (\mu_{\mathcal{N}}^2 + \omega \mathcal{L})\mathbf{I}^v \\ r\mathbf{I}^h \end{pmatrix}. \tag{9}$$

Here, we use $[\mathbf{xy}]$ as a short hand to denote a vector whose i^{th} component is given by $x_i y_i$. In $\mathcal{F}$ and $\mathcal{V}$, compartments 1 to $\mathcal{N}^2$ refer respectively to exposed vectors in sites 1 through $\mathcal{N}^2$, compartments $\mathcal{N}^2+1$ to $2\mathcal{N}^2$ refer respectively to exposed hosts in sites 1 through $\mathcal{N}^2$, compartments $2\mathcal{N}^2+1$ through $3\mathcal{N}^2$ refer respectively to infectious vectors in sites 1 through $\mathcal{N}^2$, and components $3\mathcal{N}^2+1$ through $4\mathcal{N}^2$ refer respectively to the infectious hosts in sites 1 through $\mathcal{N}^2$. We note that we have defined $\mathcal{F}$ to be the rate and which new *infectious* hosts and vectors are generated, rather than the more standard convention where $\mathcal{F}$ represents the rate at which *newly exposed but not yet infectious* hosts and vectors are generated. Our convention allows us to focus on the spatial spread of agents who are actually able to propagate the disease. In the subsection which follows our derivation of the second generation matrices and $\mathcal{R}_0$ eigenvalue problem, we show that the choice between the two conventions is immaterial for determining the basic reproduction number.

The matrices F and V are defined to be the Jacobian matrices of $\mathcal{F}$ and $\mathcal{V}$ taken about the disease-free equilibrium. We find the following:

$$F = \begin{pmatrix} 0_{2\mathcal{N}^2} & 0_{2\mathcal{N}^2} \\ F_{21} & 0_{2\mathcal{N}^2} \end{pmatrix}, \tag{10}$$

$$V = \begin{pmatrix} V_{11} & V_{12} \\ 0_{2\mathcal{N}^2} & V_{22} \end{pmatrix}, \tag{11}$$

where the the $2\mathcal{N}^2$ dimensional square sub-matrices $F_{21}, V_{11}, V_{12}, V_{22}$ are defined by

$$F_{21} = \begin{pmatrix} p^v 1_{\mathcal{N}^2} & 0_{\mathcal{N}^2} \\ 0_{1\mathcal{N}^2} & p^h 1_{\mathcal{N}^2} \end{pmatrix}, \tag{12}$$

$$V_{11} = \begin{pmatrix} p^v 1_{\mathcal{N}^2} + \mu_{\mathcal{N}}^2 + \omega \mathcal{L} & 0_{\mathcal{N}^2} \\ 0_{1\mathcal{N}^2} & p^h 1_{\mathcal{N}^2} \end{pmatrix}, \tag{13}$$

$$V_{21} = \begin{pmatrix} 0_{1\mathcal{N}^2} & -\beta^v b \frac{1}{N^h} N_{\mathcal{N}^2}^{ve} \\ -\beta^h b 1_{\mathcal{N}^2} & 0_{1\mathcal{N}^2} \end{pmatrix}, \tag{14}$$

and

$$V_{22} = \begin{pmatrix} \mu_{\mathcal{N}}^2 + \omega \mathcal{L} & 0_{\mathcal{N}^2} \\ 0_{1\mathcal{N}^2} & r 1_{\mathcal{N}^2} \end{pmatrix}. \tag{15}$$

Note that we use $N^{ve}_{\mathcal{N}^2}$ to denote the $\mathcal{N}^2 \times \mathcal{N}^2$ diagonal matrix with diagonal entries N_i^{ve} defined in Eq. (7). The inverse V^{-1} is given by

$$V^{-1} = \begin{pmatrix} V_{11}^{-1} & -V_{11}^{-1} V_{12} V_{22}^{-1} \\ 0_{2\mathcal{N}^2} & V_{22} \end{pmatrix}. \tag{16}$$

The next generation matrix FV^{-1} is thus given by:

$$FV^{-1} = \begin{pmatrix} 0_{2\mathcal{N}^2} & 0_{2\mathcal{N}^2} \\ F_{21} V_{11}^{-1} & -F_{21} V_{11}^{-1} V_{12} V_{22}^{-1} \end{pmatrix}, \tag{17}$$

where $F_{21} V_{11}^{-1}$ and $-F_{21} V_{11}^{-1} V_{12} V_{22}^{-1}$ are given by

$$F_{21} V_{11}^{-1} = \begin{pmatrix} p^v \left(p^v 1_{\mathcal{N}^2} + \mu_{\mathcal{N}^2} + \omega \mathcal{L}\right)^{-1} & 0_{\mathcal{N}^2} \\ 0_{\mathcal{N}^2} & 1_{\mathcal{N}^2} \end{pmatrix}, \tag{18}$$

and

$$-F_{21} V_{11}^{-1} V_{12} V_{22}^{-1} = \begin{pmatrix} 0_{\mathcal{N}^2} & p^v \left(p^v 1_{\mathcal{N}^2} + \mu_{\mathcal{N}^2} + \omega \mathcal{L}\right)^{-1} \beta^v \frac{b}{r} (N^h)^{-1} N^{ve}_{\mathcal{N}^2} \\ \beta^h b \left(\mu_{\mathcal{N}^2} + \omega \mathcal{L}\right)^{-1} & 0_{\mathcal{N}^2} \end{pmatrix}. \tag{19}$$

If $\mathbf{E}^v$, $\mathbf{E}^h$, $\mathbf{I}^v$, and $\mathbf{I}^h$ exposed and infectious vectors and hosts are introduced as a perturbation to a completely susceptible disease-free equilibrium state, the distribution of newly generated exposed and infectious vectors will be given by the next generation matrix's action on the $4\mathcal{N}^2$ dimensional column matrix $((\mathbf{E}^v)^T, (\mathbf{E}^h)^T, (\mathbf{I}^v)^T, (\mathbf{I}^h)^T)^T$ (where the superscript 'T' denotes the matrix transpose) as follows:

$$\begin{aligned} FV^{-1} \begin{pmatrix} \mathbf{E}^v \\ \mathbf{E}^h \\ \mathbf{I}^v \\ \mathbf{I}^h \end{pmatrix} &= \begin{pmatrix} \mathbf{0} \\ \mathbf{0} \\ p^v \left(p^v 1_{\mathcal{N}^2} + \mu_{\mathcal{N}^2} + \omega \mathcal{L}\right)^{-1} \mathbf{E}^v \\ \mathbf{E}^h \end{pmatrix} \\ &\quad + \begin{pmatrix} \mathbf{0} \\ \mathbf{0} \\ p^v \left(p^v 1_{\mathcal{N}^2} + \mu_{\mathcal{N}^2} + \omega \mathcal{L}\right)^{-1} \beta^v \frac{b}{r} (N^h)^{-1} N^{ve}_{\mathcal{N}^2} \mathbf{I}^h \\ \beta^h b \left(\mu_{\mathcal{N}^2} + \omega \mathcal{L}\right)^{-1} \mathbf{I}^v \end{pmatrix}. \end{aligned} \tag{20}$$

The eigenvalues λ of the next generation matrix can be found by solving the following characteristic equation:

$$\begin{aligned} 0 &= \mathrm{Det}\left[\lambda 1_{4\mathcal{N}^2} - FV^{-1}\right] \\ &= \lambda^{2\mathcal{N}^2} \mathrm{Det}\left[\lambda^2 1_{\mathcal{N}^2} - \beta^v b \, \beta^h \frac{b}{r} p^v \left(\mu_{\mathcal{N}^2} + \omega \mathcal{L}\right)^{-1} \left(p^v 1_{\mathcal{N}^2} + \mu_{\mathcal{N}^2} + \omega \mathcal{L}\right)^{-1} (N^h)^{-1} N^{ve}_{\mathcal{N}^2}\right]. \end{aligned} \tag{21}$$

Letting $\mathcal{M}$ denote the following matrix

$$\mathcal{M} = \beta^h b \ \left(\mu_{\mathcal{N}^2} + \omega \mathcal{L}\right)^{-1} p^v \left(p^v 1_{\mathcal{N}^2} + \mu_{\mathcal{N}^2} + \omega \mathcal{L}\right)^{-1} \beta^v \frac{b}{r} (N^h)^{-1} N^{ve}_{\mathcal{N}^2}, \tag{22}$$

the above characteristic equation can be written more compactly:

$$0 = \lambda^{2\mathcal{N}^2} \mathrm{Det}\left[\lambda^2 1_{\mathcal{N}^2} - \mathcal{M}\right]. \tag{23}$$

The corresponding eigenvectors of FV^{-1} are determined by the relation

$$\lambda \begin{pmatrix} \mathbf{0} \\ \mathbf{0} \\ \mathbf{I}^v \\ \mathbf{I}^h \end{pmatrix} = \begin{pmatrix} \mathbf{0} \\ \mathbf{0} \\ p^v \left(p^v 1_{\mathcal{N}^2} + \mu_{\mathcal{N}^2} + \omega \mathcal{L}\right)^{-1} \beta^v \frac{b}{r} (N^h)^{-1} N^{ve}_{\mathcal{N}^2} \mathbf{I}^h \\ \beta^h b \left(\mu_{\mathcal{N}^2} + \omega \mathcal{L}\right)^{-1} \mathbf{I}^v \end{pmatrix}. \tag{24}$$

Note that the eigenvalues of $(FV^{-1})^2$ are given by the set $\{\lambda^2 | \, \lambda \text{ an eigenvalue of } FV^{-1}\}$, with corresponding eigenvectors determined by

$$\lambda^2 \begin{pmatrix} \mathbf{0} \\ \mathbf{0} \\ \mathbf{I}^v \\ \mathbf{I}^h \end{pmatrix} = \begin{pmatrix} \mathbf{0} \\ \mathbf{0} \\ \underline{\mathcal{M}}\mathbf{I}^v \\ \mathcal{M}\mathbf{I}^h \end{pmatrix}, \tag{25}$$

where

$$\underline{\mathcal{M}} = p^v \left(p^v 1_{\mathcal{N}^2} + \mu_{\mathcal{N}^2} + \omega\mathcal{L}\right)^{-1} \beta^v \frac{b}{r} \left(N^h\right)^{-1} N^{ve}_{\mathcal{N}^2} \beta^h b \left(\mu_{\mathcal{N}^2} + \omega\mathcal{L}\right)^{-1}. \tag{26}$$

The matrices $\mathcal{M}$ and $\underline{\mathcal{M}}$ have identical spectra (this follows from the property that AB has the same spectrum of BA for any two square matrices A and B), and so we see that the eigenvalues λ of the next generation matrix can also be determined by solving

$$0 = \lambda^{2\mathcal{N}^2} \text{Det}\left[\lambda^2 1_{\mathcal{N}^2} - \underline{\mathcal{M}}\right]. \tag{27}$$

We refer to $\underline{\mathcal{M}}$ and $\mathcal{M}$ as the "second generation" matrices.

The basic reproduction number, $\mathcal{R}_0$, is defined as the spectral radius of FV^{-1}. The next generation matrix is a non-negative matrix, so $\mathcal{R}_0$ is, itself, an eigenvalue of FV^{-1} such that all other eigenvalues are no greater in magnitude [2]. Furthermore, to the eigenvalue $\mathcal{R}_0$, there corresponds eigenvector(s) with all non-negative components [2], and these will be the only biologically relevant eigenvectors of interest (there is no biological meaning to a negative or complex valued distribution of hosts or vectors). The non-negative eigenvectors corresponding to $\mathcal{R}_0$ represent the worst case scenario initial distribution of infected hosts and vectors which produces the largest asymptotic infected growth rate under the disease dynamics linearized about the disease-free equilibrium; e.g. if one introduces a given total number of infected vectors and hosts into a disease-free equilibrium system according to some chosen distribution, choosing a distribution which corresponds to a non-negative eigenvector with eigenvalue $\mathcal{R}_0$ will cause disease levels to grow as rapidly as possible (ignoring short-lived transient effects). Equations (24) and (25) show that these worst case scenario distributions will be comprised entirely of infectious (as opposed to exposed) vectors and hosts. We do not use the next generation matrix itself in our analysis, but instead utilize the second generation matrices to determine $\mathcal{R}_0$ by finding the largest non-negative solutions to either Eq. (23) or Eq. (27), and we find the corresponding worst case scenario spatial distributions of infectious host and vectors by solving the following eigenvector equations:

$$\mathcal{R}_0^2 \mathbf{I}^h = \mathcal{M}\mathbf{I}^h \tag{28}$$

$$\mathcal{R}_0^2 \mathbf{I}^v = \underline{\mathcal{M}}\mathbf{I}^v. \tag{29}$$

3.1 Alternative more standard convention

Under the more standard convention, the new infection vector $\mathcal{F}^*$ and the transfer vector $\mathcal{V}^*$ are defined as

$$\mathcal{F}^* = \begin{pmatrix} \beta^v b \frac{1}{N^h} [\mathbf{S}^v \mathbf{I}^h] \\ \beta^h b \frac{1}{N^h} [\mathbf{S}^h \mathbf{I}^v] \\ \mathbf{0} \\ \mathbf{0} \end{pmatrix}, \tag{30}$$

and

$$\mathcal{V}^* = \begin{pmatrix} (p^v 1_{\mathcal{N}^2} + \mu^2_{\mathcal{N}} + \omega\mathcal{L})\mathbf{E}^v \\ p^h \mathbf{E}^h \\ (\mu^2_{\mathcal{N}} + \omega\mathcal{L})\mathbf{I}^v - p^v \mathbf{E}^v \\ r\mathbf{I}^h - p^h \mathbf{E}^h \end{pmatrix}. \tag{31}$$

The corresponding next generation matrix F^*V^{*-1} can be shown to be

$$F^*V^{*-1} = \begin{pmatrix} -F^*_{12}V^{*-1}_{22}V^*_{21}V^{*-1}_{11} & F^*_{12}V^{*-1}_{22} \\ 0_{2\mathcal{N}^2} & 0_{2\mathcal{N}^2} \end{pmatrix}, \tag{32}$$

where

$$-F^*_{12}V^{*-1}_{22}V^*_{21}V^{*-1}_{11} = \begin{pmatrix} 0_{\mathcal{N}^2} & \beta^v \frac{b}{r}(N^h)^{-1}N^{ve}_{\mathcal{N}^2} \\ \beta^h b\left(\mu_{\mathcal{N}^2}+\omega\mathcal{L}\right)^{-1} p^v \left(p^v 1_{\mathcal{N}^2}+\mu_{\mathcal{N}^2}+\omega\mathcal{L}\right)^{-1} & 0_{\mathcal{N}^2} \end{pmatrix}, \tag{33}$$

and

$$F^*_{12}V^{*-1}_{22} = \begin{pmatrix} 0_{\mathcal{N}^2} & \beta^v \frac{b}{r}(N^h)^{-1}N^{ve}_{\mathcal{N}^2} \\ \beta^h b\left(\mu_{\mathcal{N}^2}+\omega\mathcal{L}\right)^{-1} & 0_{\mathcal{N}^2} \end{pmatrix}. \tag{34}$$

We thus find the action of F^*V^{*-1} on a distribution of infected hosts and vectors to be the following

$$\begin{aligned} F^*V^{*-1}\begin{pmatrix} \mathbf{E}^v \\ \mathbf{E}^h \\ \mathbf{I}^v \\ \mathbf{I}^h \end{pmatrix} &= \begin{pmatrix} \beta^v \frac{b}{r}(N^h)^{-1}N^{ve}_{\mathcal{N}^2}\mathbf{E}^h \\ \beta^h b\left(\mu_{\mathcal{N}^2}+\omega\mathcal{L}\right)^{-1} p^v \left(p^v 1_{\mathcal{N}^2}+\mu_{\mathcal{N}^2}+\omega\mathcal{L}\right)^{-1}\mathbf{E}^v \\ \mathbf{0} \\ \mathbf{0} \end{pmatrix} \\ &\quad + \begin{pmatrix} \beta^v \frac{b}{r}(N^h)^{-1}N^{ve}_{\mathcal{N}^2}\mathbf{I}^h \\ \beta^h b\left(\mu_{\mathcal{N}^2}+\omega\mathcal{L}\right)^{-1}\mathbf{I}^v \\ \mathbf{0} \\ \mathbf{0} \end{pmatrix}. \end{aligned} \tag{35}$$

The eigenvalue problem for F^*V^{*-1} is given by

$$\lambda\begin{pmatrix} \mathbf{E}^v \\ \mathbf{E}^h \\ \mathbf{0} \\ \mathbf{0} \end{pmatrix} = \begin{pmatrix} \beta^v \frac{b}{r}(N^h)^{-1}N^{ve}_{\mathcal{N}^2}\mathbf{E}^h \\ \beta^h b\left(\mu_{\mathcal{N}^2}+\omega\mathcal{L}\right)^{-1} p^v \left(p^v 1_{\mathcal{N}^2}+\mu_{\mathcal{N}^2}+\omega\mathcal{L}\right)^{-1}\mathbf{E}^v \\ \mathbf{0} \\ \mathbf{0} \end{pmatrix}. \tag{36}$$

The eigenvalue problem for $(F^*V^{*-1})^2$ is thus found to be

$$\lambda^2\begin{pmatrix} \mathbf{E}^v \\ \mathbf{E}^h \\ \mathbf{0} \\ \mathbf{0} \end{pmatrix} = \begin{pmatrix} \underline{\mathcal{M}}^*\mathbf{E}^v \\ \mathcal{M}^*\mathbf{E}^h \\ \mathbf{0} \\ \mathbf{0} \end{pmatrix}, \tag{37}$$

where

$$\underline{\mathcal{M}}^* = \beta^v \frac{b}{r}(N^h)^{-1}N^{ve}_{\mathcal{N}^2}\beta^h b\left(\mu_{\mathcal{N}^2}+\omega\mathcal{L}\right)^{-1} p^v \left(p^v 1_{\mathcal{N}^2}+\mu_{\mathcal{N}^2}+\omega\mathcal{L}\right)^{-1}, \tag{38}$$

and

$$\mathcal{M}^* = \beta^h b\left(\mu_{\mathcal{N}^2}+\omega\mathcal{L}\right)^{-1} p^v \left(p^v 1_{\mathcal{N}^2}+\mu_{\mathcal{N}^2}+\omega\mathcal{L}\right)^{-1}\beta^v \frac{b}{r}(N^h)^{-1}N^{ve}_{\mathcal{N}^2}. \tag{39}$$

Comparing the above expressions to Eqs. (22) and (26), we see that $\mathcal{M}^* = \mathcal{M}$, and that $\underline{\mathcal{M}}^*$ and $\underline{\mathcal{M}}$ have identical specta, and thus conclude that $\mathcal{M}^*, \mathcal{M}, \underline{\mathcal{M}}^*$, and $\underline{\mathcal{M}}$ all all have identical spectra and have a largest eigenvalue $\mathcal{R}_0^2$. The second generation matrices under this more alternative more common convention indicate the spatial propagation of exposed but not infectious host and vectors, while our convention indicates the spatial propagation of infectious hosts and vectors.

4 Special case analytic eigenvalue and eigenvector solutions

4.1 Isolated sites

For a neighborhood comprised of a single isolated site (e.g. $\mathcal{N}^2 = 1$), the hopping rate ω and Laplacian matrix $\mathcal{L}$ are irrelevant, and our disease system reduces to a basic single-patch SEIR model. In this case, the characteristic equations in (23) and (27) are equivalent and trivial to solve, and we find

$$\mathcal{R}_0 = \begin{cases} \mathcal{R}_{00}, & \text{Uncontrolled isolated site} \\ \mathcal{R}_{0N}, & \text{Non-compliant isolated site} \\ \mathcal{R}_{0C}, & \text{Compliant isolated site,} \end{cases} \tag{40}$$

where the single-site uncontrolled, non-compliant, and compliant basic reproduction numbers are defined, respectively, as

$$\begin{aligned} \mathcal{R}_{00} &= \sqrt{\beta^v \frac{b}{r} \beta^h \frac{b}{\mu_0} \frac{p_v}{p_v + \mu_0} \frac{1}{N^h} \frac{\Lambda_0}{\mu_0}} \\ \mathcal{R}_{0N} &= \sqrt{\beta^v \frac{b}{r} \beta^h \frac{b}{\mu_N} \frac{p_v}{p_v + \mu_N} \frac{1}{N^h} \frac{\Lambda_N}{\mu_N}} \\ \mathcal{R}_{0C} &= \sqrt{\beta^v \frac{b}{r} \beta^h \frac{b}{\mu_C} \frac{p_v}{p_v + \mu_C} \frac{1}{N^h} \frac{\Lambda_C}{\mu_C}}. \end{aligned} \tag{41}$$

4.2 Homogeneous systems

For the special cases of no control, 100% compliance, and 100% non-compliance, all model parameters are equivalent at every site in the neighborhood. Specifically, the diagonal death rate matrix $\mu_{\mathcal{N}^2}$ and emergence rate vector $\mathbf{\Lambda}$ are given by the following:

$$\mu_{\mathcal{N}^2} = \begin{cases} \mu_0 1_{\mathcal{N}^2}, & \text{No controls applied to system} \\ \mu_N 1_{\mathcal{N}^2}, & \text{Controlled system, 100\% non-compliance} \\ \mu_C 1_{\mathcal{N}^2}, & \text{Controlled system, 100\% compliance,} \end{cases} \tag{42}$$

$$\mathbf{\Lambda} = \begin{cases} \Lambda_0 \mathbf{1}, & \text{No controls applied to system} \\ \Lambda_N \mathbf{1}, & \text{Controlled system, 100\% non-compliance} \\ \Lambda_C \mathbf{1}, & \text{Controlled system, 100\% compliance.} \end{cases} \tag{43}$$

Multiplying both sides of Eq. (7) by the matrix $(\mu_{\mathcal{N}^2} + \omega\mathcal{L})$, the equilibrium vector population $\mathbf{N}^{ve}$ for homogeneous systems is found to be

$$\mathbf{N}^{ve} = \begin{cases} \frac{\Lambda_0}{\mu_0}\mathbf{1}, & \text{No controls applied to system} \\ \frac{\Lambda_N}{\mu_N}\mathbf{1}, & \text{Controlled system, 100\% non-compliance} \\ \frac{\Lambda_C}{\mu_C}\mathbf{1}, & \text{Controlled system, 100\% compliance.} \end{cases} \tag{44}$$

The homogeneous system second generation matrices are given by

$$\begin{aligned} \mathcal{M} &= \underline{\mathcal{M}} \\ &= \begin{cases} \mathcal{R}_{00}^2 \left(1_{\mathcal{N}^2} + \frac{\omega}{\mu_0}\mathcal{L}\right)^{-1} \left(1_{\mathcal{N}^2} + \frac{\omega}{p^v + \mu_0}\mathcal{L}\right)^{-1}, & \text{Uncontrolled system} \\ \mathcal{R}_{0N}^2 \left(1_{\mathcal{N}^2} + \frac{\omega}{\mu_N}\mathcal{L}\right)^{-1} \left(1_{\mathcal{N}^2} + \frac{\omega}{p^v + \mu_N}\mathcal{L}\right)^{-1}, & \text{100\% non-compliant system} \\ \mathcal{R}_{0C}^2 \left(1_{\mathcal{N}^2} + \frac{\omega}{\mu_C}\mathcal{L}\right)^{-1} \left(1_{\mathcal{N}^2} + \frac{\omega}{p^v + \mu_C}\mathcal{L}\right)^{-1}, & \text{100\% compliant system,} \end{cases} \end{aligned} \tag{45}$$

where $\mathcal{R}_{00}$, $\mathcal{R}_{0N}$, and $\mathcal{R}_{0C}$ are the single-site basic reproduction numbers defined in Eq. (41).

To find $\mathcal{R}_0$, consider first the case of no controls applied to the system, for which the characteristic equation (23) reduces to

$$0 \quad = \quad \lambda^{2\mathcal{N}^2} \mathrm{Det}\left[\lambda^2 1_{\mathcal{N}^2} - \mathcal{R}_{00}^2 \left(1_{\mathcal{N}^2} + \frac{\omega}{p^v + \mu_0}\mathcal{L}\right)^{-1} \left(1_{\mathcal{N}^2} + \frac{\omega}{\mu_0}\mathcal{L}\right)^{-1}\right], \quad (46)$$

where $\mathcal{R}_{00}$ is the single-site uncontrolled basic reproduction number defined in Eq. (41). The non-zero eigenvalues of FV^{-1} can be found by using the properties of determinants to manipulate Eq. (46) into and solving the following alternative characteristic equation for the eigenvalues z of the matrix $\mathcal{K}$:

$$0 \quad = \quad \mathrm{Det}\left[z 1_{\mathcal{N}^2} - \mathcal{K}\right], \quad (47)$$

where we define

$$\mathcal{K} \quad = \quad \frac{\omega}{\mu_0}\mathcal{L} + \frac{\omega}{p^v + \mu_0}\mathcal{L} + \frac{\omega^2}{(p^v + \mu_0)\mu_0}\mathcal{L}^2. \quad (48)$$

and where the eigenvalues z of $\mathcal{K}$ are related to the non-zero eigenvalues λ of FV^{-1} by

$$z \quad = \quad -1 + \frac{\mathcal{R}_{00}^2}{\lambda^2}. \quad (49)$$

The Laplacian is a symmetric positive semi-definite matrix [3], and so the matrix $\mathcal{K}$ is also symmetric and positive semi-definite [4]. Therefore, the eigenvalues z of $\mathcal{K}$ must be real and non-negative [4], and this fact together with Eq. (49) implies that the non-zero eigenvalues λ of FV^{-1} must be real and satisfy

$$0 < |\lambda| \leq \mathcal{R}_{00}. \quad (50)$$

Thus, if FV^{-1} has $\pm\mathcal{R}_{00}$ for an eigenvalue, then its spectral radius will be given by $\mathcal{R}_{00}$. Equation (49) shows that $\lambda = \pm\mathcal{R}_{00}$ is an eigenvalue of FV^{-1} if and only if $z = 0$ is a eigenvalue of $\mathcal{K}$, and $z = 0$ is an eigenvalue of $\mathcal{K}$ if and only if $\mathrm{Det}\left[\mathcal{K}\right] = 0$. Equation (48) shows that $\mathrm{Det}\left[\mathcal{K}\right]$ will be proportional to $\mathrm{Det}\left[\mathcal{L}\right]$, and the fact that all Laplacian matrices have zero for an eigenvalue [3] implies that $\mathrm{Det}\left[\mathcal{L}\right] = 0$. We thus conclude that $\mathrm{Det}\left[\mathcal{K}\right] = 0$ and that $\mathcal{R}_{00}$ is the spectral radius of FV^{-1} for a homogeneous uncontrolled system. Analogous arguments can be made for homogeneous 100% non-compliant and compliant controlled systems, from which we obtain the following result.

$$\mathcal{R}_0 \quad = \quad \begin{cases} \mathcal{R}_{00}, & \text{Uncontrolled system} \\ \mathcal{R}_{0N}, & \text{100\% non-compliant system} \\ \mathcal{R}_{0C}, & \text{100\% compliant system.} \end{cases} \quad (51)$$

Inputting Eqs. (45) and (51) into the eigenvector equations (28) and (29), and utilizing the fact that $\mathcal{L}\mathbf{1} = \mathbf{0}$ for any Laplacian matrix, the normalized eigenvectors $\mathbf{I}^h$ and $\mathbf{I}^v$ of the second generation matrices corresponding to the eigenvalue $\mathcal{R}_0^2$, regardless of control or compliance, are found to be

$$\begin{aligned} \mathbf{I}^h \quad &= \quad \mathbf{I}^v \\ &= \quad \frac{1}{\mathcal{N}^2}\mathbf{1}. \end{aligned} \quad (52)$$

4.3 Infinitely fast hopping

For the case of infinitely rapid mosquito hopping, we consider the limit $\omega \to \infty$. This is a singular limit due to the fact that the fact that the matrices $(\mu_{\mathcal{N}^2} + \omega\mathcal{L})^{-1}$ and $(p^v 1_{\mathcal{N}^2} + \mu_{\mathcal{N}^2} + \omega\mathcal{L})^{-1}$ diverge and become ill-defined as $\omega \to \infty$, and so mathematical analysis must be handled with some care. Here, we utilize the methods of Tien et al. outlined in [5]: we consider the quantities μ_0/ω and $(\mu_0 + p^v)/\omega$ to be small parameters, perform Laurent expansions of $(\mu_{\mathcal{N}^2} + \omega\mathcal{L})^{-1}$ and $(p^v 1_{\mathcal{N}^2} + \mu_{\mathcal{N}^2} + \omega\mathcal{L})^{-1}$ about these small parameters, respectively, and retain the lowest order terms of the expansions (order negative one) as matrix expressions valid in the $\omega \to \infty$ limit. We give only the results of this process and use them to obtain an expression for $\mathcal{R}_0$ in the $\omega \to \infty$ limit, and refer the reader to [5] for details on performing the Laurent expansions.

Following [5], we obtain the following in the infinitely fast hopping limit:

$$(\mu_{\mathcal{N}^2} + \omega\mathcal{L})^{-1} \underset{\omega\to\infty}{=} \frac{\mathbf{1}\mathbf{1}^T}{\mathcal{N}^2 \langle\mu\rangle} \tag{53}$$

$$(p^v 1_{\mathcal{N}^2} + \mu_{\mathcal{N}^2} + \omega\mathcal{L})^{-1} \underset{\omega\to\infty}{=} \frac{\mathbf{1}\mathbf{1}^T}{\mathcal{N}^2 (p^v + \langle\mu\rangle)}, \tag{54}$$

where the expression $\langle g\rangle$ denotes the average of a site dependent quantity g over the entire neighborhood, and the product $\mathbf{1}\mathbf{1}^T$ is the $\mathcal{N}^2 \times \mathcal{N}^2$ matrix filled entirely with ones. Equations (7) and (53) yield the following equilibrium vector population:

$$\mathbf{N}^{ve} \underset{\omega\to\infty}{=} \frac{\langle\Lambda\rangle}{\langle\mu\rangle}\mathbf{1}. \tag{55}$$

Similarly, the second generation matrices defined in Eqs. (22) and (26) reduce to the following:

$$\begin{aligned} \mathcal{M} &\underset{\omega\to\infty}{=} \underline{\mathcal{M}} \\ &\underset{\omega\to\infty}{=} \beta^v \frac{b}{r}\beta^h \frac{b}{\langle\mu\rangle}\frac{p^v}{p^v + \langle\mu\rangle}\frac{1}{N^h}\frac{\langle\Lambda\rangle}{\langle\mu\rangle}\frac{\mathbf{1}\mathbf{1}^T}{\mathcal{N}^2}. \end{aligned} \tag{56}$$

The above matrix is comprised of $\mathcal{N}^2$ identical rows, so it has rank 1, and thus has only one non-zero eigenvalue (with algebraic multiplicity one) [4]. The matrix trace gives the sum of all eigenvalues for any square matrix, so the single non-zero eigenvalue of $\mathcal{M}$ (or equivalently $\underline{\mathcal{M}}$) is found by taking the trace of $\mathcal{M}$, and $\mathcal{R}_0$ will be given by the positive square root of the result. From Eq. (56), we find

$$\mathcal{R}_0 \underset{\omega\to\infty}{=} \sqrt{\beta^v \frac{b}{r}\beta^h \frac{b}{\langle\mu\rangle}\frac{p^v}{p^v + \langle\mu\rangle}\frac{1}{N^h}\frac{\langle\Lambda\rangle}{\langle\mu\rangle}}. \tag{57}$$

We thus have $\mathcal{M} = \underline{\mathcal{M}} = \mathcal{R}_0^2 \mathbf{1}\mathbf{1}^T/\mathcal{N}^2$, and the normalized eigenvector solutions to Eqs. (28) and (29) are found to be

$$\begin{aligned} \mathbf{I}^h &\underset{\omega\to\infty}{=} \mathbf{I}^v \\ &\underset{\omega\to\infty}{=} \frac{1}{\mathcal{N}^2}\mathbf{1}. \end{aligned} \tag{58}$$

4.4 Infinitely slow hopping

In the infinitely slow hopping $\omega = 0$ limit, all patches decouple from one another. This is not a singular limit, and we can find expressions for the equilibrium vector population and the second generation matrices by setting $\omega = 0$ in Eqs. (7), (22), and (26):

$$\mathbf{N}^{ve}_{(0)} = \mu^{-1}_{\mathcal{N}^2}\mathbf{\Lambda}, \tag{59}$$

$$\begin{aligned}\mathcal{M}_{(0)} &= \underline{\mathcal{M}}_{(0)} \\ &= \mathrm{Diag}\left\{\beta^h \frac{b}{\mu_i}\beta^v \frac{b}{r}\frac{p_v}{p_v+\mu_i}\frac{1}{N^h}\frac{\Lambda_i}{\mu_i}\right\}.\end{aligned} \tag{60}$$

Here and throughout the rest of this paper, the subscript (0) refers to quantities evaluated for $\omega = 0$. The second generation matrices are diagonal in the no-hopping limit, and the spectral radii are consequently trivial to find:

$$\begin{aligned}\mathcal{R}_{0(0)} &= \max_i \left\{\sqrt{\beta^h \frac{b}{\mu_i}\beta^v \frac{b}{r}\frac{p^v}{p^v+\mu_i}\frac{1}{N^h}\frac{\Lambda_i}{\mu_i}}\right\} \\ &= \begin{cases}\mathcal{R}_{00}, & \text{Uncontrolled system}\\ \mathcal{R}_{0N}, & \text{Less than 100\% compliant}\\ \mathcal{R}_{0C}, & \text{100\% compliant.}\end{cases}\end{aligned} \tag{61}$$

The eigenspaces of $\mathcal{M}_{(0)}$ and $\underline{\mathcal{M}}_{(0)}$ corresponding to $\mathcal{R}_0^2$ are degenerate: any vector which is distributed entirely within any subset of the non-compliant sites will be an eigenvector with eigenvalue $\mathcal{R}_0^2$ (degeneracy vanishes only for the special case of a spatially homogeneous system). Consequently, we know that the worst case distributions of infectious vectors and hosts will lie entirely within the non-compliant sites in the no-hopping limit, but we have no way of distinguishing which, if any, of all possible eigenvectors are more "important" or "correct" practically, in terms of biological meaning and control strategies. This ambiguity can be resolved to some extent by considering perturbations to the no-hopping case caused by small but non-zero hopping rates.

5 Perturbation theory for finitely slow hopping rates

For the case of slow but non-zero hopping, we analyze our system using degenerate perturbation theory. Here, we give the necessary definitions, results, and interpretations. The details of the perturbation formalism can be found at a utilitarian level in Ref. [6], or at a more rigorous mathematical level in Ref. [7]. Perturbation theory will provide accurate results for our system when the parameters ω/μ_0 and $\omega/(\mu_0 + p^v)$ are much smaller than unity. Under this assumption, one can find simplified approximate expressions for $\mathbf{N}^{ve}, \mathcal{M}$, and $\underline{\mathcal{M}}$ by writing the inverse matrices in Eqs. (7), (22), and (26) as Taylor expansions in ω, and retaining only the zeroth and first order terms. We define the perturbations $\delta\mathbf{N}^{ve}, \delta\mathcal{M}$, and $\delta\underline{\mathcal{M}}$ through the following equations:

$$\begin{aligned}\mathbf{N}^{ve} &= \mathbf{N}^{ve}_{(0)} + \delta\mathbf{N}^{ve} \\ \mathcal{M} &= \mathcal{M}_{(0)} + \delta\mathcal{M} \\ \underline{\mathcal{M}} &= \underline{\mathcal{M}}_{(0)} + \delta\underline{\mathcal{M}},\end{aligned} \tag{62}$$

where the zeroth order unperturbed terms are defined as the no-hopping limit expressions in Eqs. (59) and (60), and the perturbations are linear in ω. The perturbations are given explicitly by the following formulas:

$$\delta\mathbf{N}^{ve} = -\omega\mu_{\mathcal{N}^2}^{-1}\mathcal{L}\mu_{\mathcal{N}^2}^{-1}\mathbf{\Lambda}, \tag{63}$$

$$\begin{aligned}\delta\mathcal{M} = {} & \beta^h b\mu_{\mathcal{N}^2}^{-1}\beta^v \frac{b}{r}p^v(p^v 1_{\mathcal{N}^2} + \mu_{\mathcal{N}^2})^{-1}(N^h)^{-1}\delta N^{ve}_{\mathcal{N}^2} - \omega\beta^v \frac{b}{r}\beta^v b\mu_{\mathcal{N}^2}^{-1} \\ & \times\left(\mathcal{L}\mu_{\mathcal{N}^2}^{-1} + (p^v 1_{\mathcal{N}^2} + \mu_{\mathcal{N}^2})^{-1}\mathcal{L}\right)p^v(p^v 1_{\mathcal{N}^2} + \mu_{\mathcal{N}^2})^{-1}(N^h)^{-1}N^{ve}_{\mathcal{N}^2(0)}.\end{aligned} \tag{64}$$

$$\delta\underline{\mathcal{M}} = \beta^h b\mu_{\mathcal{N}^2}^{-1}\beta^v\frac{b}{r}p^v(p^v 1_{\mathcal{N}^2}+\mu_{\mathcal{N}^2})^{-1}(N^h)^{-1}\delta N_{\mathcal{N}^2}^{ve} - \omega\beta^v\frac{b}{r}p^v(p^v 1_{\mathcal{N}^2}+\mu_{\mathcal{N}^2})^{-1} \times\left(\mathcal{L}(p^v 1_{\mathcal{N}^2}+\mu_{\mathcal{N}^2})^{-1}(N^h)^{-1}N_{\mathcal{N}^2(0)}^{ve} + (N^h)^{-1}N_{\mathcal{N}^2(0)}^{ve}\mu_{\mathcal{N}^2}^{-1}\mathcal{L}\right)\beta^v b\mu_{\mathcal{N}^2}^{-1}, \tag{65}$$

where $N_{\mathcal{N}^2(0)}^{ve}$ denotes the diagonal matrix with diagonal entries given by the components of $\mathbf{N}_{(0)}^{ve}$, and $\delta N_{\mathcal{N}^2}^{ve}$ denotes the diagonal matrix with diagonal entries given by the components of $\delta\mathbf{N}^{ve}$. Similarly, we define the perturbation to the basic reproduction number through the following decomposition

$$\mathcal{R}_0 = \mathcal{R}_{0(0)} + \delta\mathcal{R}_0, \tag{66}$$

where $\mathcal{R}_{0(0)}$ is the no-hopping basic reproduction number given in Eq. (61), and where $\delta\mathcal{R}_0$ is linear in ω. The perturbed basic reproduction number squared is the spectral radius of the perturbed second generation matrices. It and the corresponding perturbed eigenvectors of $\mathcal{M}$ and $\underline{\mathcal{M}}$ are defined by the following perturbed eigenvalue problems, where terms greater than first order in ω are ignored:

$$(\mathcal{R}_{0(0)}+\delta\mathcal{R}_0)^2(\mathbf{I}_{(0)}^h+\delta\mathbf{I}^h) = (\mathcal{M}_{(0)}+\delta\mathcal{M})(\mathbf{I}_{(0)}^h+\delta\mathbf{I}^h), \tag{67}$$
$$(\mathcal{R}_{0(0)}+\delta\mathcal{R}_0)^2(\mathbf{I}_{(0)}^v+\delta\mathbf{I}^v) = (\underline{\mathcal{M}}_{(0)}+\delta\underline{\mathcal{M}})(\mathbf{I}_{(0)}^v+\delta\mathbf{I}^v), \tag{68}$$

where $\delta\mathbf{I}^v$ and $\delta\mathbf{I}^h$ are linear in ω. In the above equations, the vectors $\mathbf{I}_{(0)}^h$ and $\mathbf{I}_{(0)}^v$ are as-of-yet undetermined members of the degenerate eigenspaces of $\mathcal{M}_{(0)}$ and $\underline{\mathcal{M}}_{(0)}$, respectively, corresponding to the eigenvalue $\mathcal{R}_{0(0)}^2$ (e.g. vectors that are distributed in some unknown manner entirely within the non-compliant sites).

Assume that exactly $J \geq 1$ sites are non-compliant, let $\{i_1, i_2, ..., i_J\}$ denote the indices of the J non-compliant sites, and let $\{\mathbf{e}_1, \mathbf{e}_2, ..., \mathbf{e}_{\mathcal{N}^2}\}$ denote the standard orthonormal basis for $\mathbb{R}^{\mathcal{N}^2}$. RStandard degenerate perturbation analysis (see Refs. [6] and [7] for details) shows that the unperturbed vectors $\mathbf{I}_{(0)}^h$ and $\mathbf{I}_{(0)}^v$, as well as the perturbations $\delta\mathcal{R}_0, \delta\mathbf{I}^v$, and $\delta\mathbf{I}^h$ are ultimately determined by the spectrum of the $J\times J$-dimensional matrix $\mathcal{W}$ defined by the following elements:

$$\mathcal{W}_{jk} = \frac{1}{2\mathcal{R}_{0N}}\mathbf{e}_{i_j}^T\,\delta\mathcal{M}\,\mathbf{e}_{i_k} = \frac{1}{2\mathcal{R}_{0N}}\delta\mathcal{M}_{i_j i_k} = \frac{1}{2\mathcal{R}_{0N}}\delta\underline{\mathcal{M}}_{i_j i_k}. \tag{69}$$

In words, $\mathcal{W}$ is formed by the matrix elements of $\delta\mathcal{M}$ (multiplied by an overall scaling factor $1/2\mathcal{R}_{0N}$) for which both indices correspond to non-compliant sites, and for our system, these elements turn out to be equivalent to the corresponding matrix elements of $\delta\underline{\mathcal{M}}$. Letting degC$(n)$ and degN(n) denote the numbers of compliant and non-compliant nearest neighbors connected to a site n, respectively, we find the following explicit expressions for the components of $\mathcal{W}$:

$$\mathcal{W}_{jk} = \begin{cases} -\kappa\,\mathrm{degN}(i_j) - \kappa\,\xi\,\mathrm{degC}(i_j), & j=k \\ \kappa, & \text{If sites } i_j \text{ and } i_k \text{ are nearest neighbors} \\ 0, & \text{otherwise,} \end{cases}$$

where the constants κ and ξ are given by

$$\kappa = \frac{1}{2}\mathcal{R}_{0N}\left[\left(\frac{\mu_N}{\mu_0}\right)^{-1} + \left(\frac{p^v}{\mu_0}+\frac{\mu_N}{\mu_0}\right)^{-1}\right]\frac{\omega}{\mu_0}, \tag{70}$$

and

$$\xi \;=\; 1+\frac{1}{1+\frac{\mu_N}{p^v+\mu_N}}\left(1-\frac{\Lambda_C/\mu_C}{\Lambda_N/\mu_N}\right). \tag{71}$$

The eigenvalues of $\mathcal{W}$ are proportional to the parameter κ, and κ is itself proportional to ω/μ_0, with the constant of proportionality determined by the non-compliant single site basic reproduction number $\mathcal{R}_{0N}$ and the ratios of the non-compliant vector death rate μ_N and extrinsic incubation period p^v to the natural death rate μ_0. The parameter ξ can vary between one and two depending on the strength of control in compliant and non-compliant sites.

Note that the matrix $-\mathcal{W}$ is diagonally dominant, symmetric, and has all real non-negative diagonally entries, and so it is positive semi-definite and thus has all real non-negative eigenvalues [8]. Let z denote the smallest of these eigenvalues. Consequently, the matrix $\mathcal{W}$ will have all real non-positive eigenvalues, the largest of which will be $-z$. Thus, the largest eigenvalue of $\mathcal{W}$ is real and non-positive. Further, because $\mathcal{W}$ has all non-positive diagonal entries and all non-negative off-diagonal entries, the matrix $\mathcal{W}+s1_J$ will be non-negative given for any non-negative constant $s \geq \max_k\{|\mathcal{W}_{kk}|\}$, where 1_J is the $J\times J$ identity matrix. Thus, by the Perron-Frobenius theorem for non-negative matrices [8], the matrix $\mathcal{W}+s1_J$ has a positive real eigenvalue λ_{PF} (the value of which depends on s) for which all other eigenvalues are less than or equal in magnitude, and for which there corresponds an eigenvector $\mathbf{x}_{PF}$ that can be taken to have all non-negative components. From these facts, we conclude that $\lambda_{PF}-s$ is the largest eigenvalue of $\mathcal{W}$ to which $\mathbf{x}_{PF}$ is a corresponding non-negative eigenvector. Thus, $\mathcal{W}$ has a largest, real, non-positive eigenvalue with a corresponding non-negative eigenvector.

Applying standard techniques from degenerate perturbation theory, the perturbation $\delta\mathcal{R}_0$ can be shown to be the largest eigenvalue of $\mathcal{W}$. From this, we conclude immediately that $\delta\mathcal{R}_0\leq 0$, and that $\delta\mathcal{R}_0$ is always proportional to κ. The J-dimensional eigenvectors $\boldsymbol{\alpha}$ defined by the relation

$$\delta\mathcal{R}_0\boldsymbol{\alpha} \;=\; \mathcal{W}\boldsymbol{\alpha}, \tag{72}$$

can be taken to be non-negative and normalized. Standard degenerate perturbation analysis shows that the components of $\boldsymbol{\alpha}$ determine $\mathbf{I}^h_{(0)}, \mathbf{I}^v_{(0)}, \delta\mathbf{I}^h$, and $\delta\mathbf{I}^v$ as follows:

$$\begin{aligned}\mathbf{I}^h_{(0)} &= \mathbf{I}^v_{(0)} \\ &= \sum_{k=1}^{J}\alpha_k\mathbf{e}_{i_k},\end{aligned} \tag{73}$$

$$\delta\mathbf{I}^h \;=\; -\sum_{k=1}^{J}\sum_{\substack{j=1\\ j\neq i_1,i_2,\ldots,i_J}}^{\mathcal{N}^2}\frac{\delta M_{j\,i_k}}{\mathcal{R}_{0N}^2-\mathcal{R}_{0C}^2}\alpha_k\mathbf{e}_{i_k}+\sum_{\substack{j=1\\ j\neq i_1,i_2,\ldots,i_J}}^{\mathcal{N}^2}\sum_{k=1}^{J}\frac{\delta M_{j\,i_k}}{\mathcal{R}_{0N}^2-\mathcal{R}_{0C}^2}\alpha_k\mathbf{e}_j, \tag{74}$$

$$\delta\mathbf{I}^v \;=\; -\sum_{k=1}^{J}\sum_{\substack{j=1\\ j\neq i_1,i_2,\ldots,i_J}}^{\mathcal{N}^2}\frac{\delta \underline{M}_{j\,i_k}}{\mathcal{R}_{0N}^2-\mathcal{R}_{0C}^2}\alpha_k\mathbf{e}_{i_k}+\sum_{\substack{j=1\\ j\neq i_1,i_2,\ldots,i_J}}^{\mathcal{N}^2}\sum_{k=1}^{J}\frac{\delta \underline{M}_{j\,i_k}}{\mathcal{R}_{0N}^2-\mathcal{R}_{0C}^2}\alpha_k\mathbf{e}_j. \tag{75}$$

The first double summations in $\delta\mathbf{I}^h$ and $\delta\mathbf{I}^v$ are the perturbations to $\mathbf{I}^h_{(0)}$ and $\mathbf{I}^v_{(0)}$ within the sites occupied by $\mathbf{I}^h_{(0)}$ and $\mathbf{I}^v_{(0)}$ (all of which are non-compliant), while the second double summations give the perturbations at the sites not occupied by $\mathbf{I}^h_{(0)}$ and

$\mathbf{I}^v_{(0)}$. The matrix element perturbations $\delta\mathcal{M}_{ij}$ and $\delta\underline{\mathcal{M}}_{ij}$ are non-zero only if $i = j$ or if site i and site j are connected, and so the eigenvector perturbations $\delta\mathbf{I}^h$ and $\delta\mathbf{I}^v$ effectively transport the unperturbed eigenvectors from connected blocks of non-compliant sites out into the corresponding bordering compliant sites. It should be noted that, due to differences in normalization schemes, the analogues of the first double summations in Eqs. (74) and (75) are not present in many of the perturbation analyses found throughout the literature. Physics applications, for example, most often normalize vectors by setting their absolute magnitudes to unity, and for our system, this more typical normalization scheme would force the perturbations $\delta\mathbf{I}^h$ and $\delta\mathbf{I}^v$ to be orthogonal to $\mathbf{I}^h_{(0)}$ and $\mathbf{I}^v_{(0)}$, thus eliminating the first double summations. The absolute magnitude normalization, however, does not have a clear biological meaning for our system. The alternative normalization scheme used in this paper has clear biological meaning in allowing $\mathbf{I}^h$ and $\mathbf{I}^v$ to represent probability distributions.

6 Relating controlled model parameters to real-world control strategies

6.1 Control strength

In our model, door-to-door adulticide and larval source reduction uniformly increase death rates and reduce emergence rates, respectively, in compliant sites only, while area-wide adulticide spray uniformly increases death rates in all sites. To door-to-door adulticide and aerial spray adulticide, we associate the *control strengths* $\rho_D \in [0, 1]$ and $\rho_A \in [0, 1]$, respectively, and to door-to-door larval source reduction, we associate the control strength $\sigma_D \in [0, 1]$. A management strategy's control strength is dependent upon both the efficacy of the strategy as well as the frequency of application, and is defined as the percent reduction in equilibrium vector population that would result from that strategy's use in an isolated unconnected neighborhood site. By this definition, for a single type of adulticide used alone at control strength ρ_i, $i \in \{D, A\}$, the increased vector death rate is given by $\mu_0/(1 - \rho_i)$, and for larval control used alone at control strength σ_D, the decreased emergence rate is given by $\Lambda_0(1 - \sigma_D)$, where μ_0 and Λ_0 are the natural death and emergence rates, respectively. Defining control strength in this way allows one to associate changes in model parameters, and therefore predictions for outbreak potential via $\mathcal{R}_0$, with notions of control effort; for high control effort, one expects large population reductions and control strengths close to 1, and for low control effort, one expects small population reductions and control strengths close to 0.

The results in Ref. [9] provide explicit expressions for the aerial spray adulticide control strength $\rho_A(f_A)$ and the door-to-door adulticide and larval control strengths $\rho_D(f_D)$ and $\sigma_D(f_D)$ written as functions of the aerial and door-to-door application frequencies f_A and f_D, and are considered to be implicit functions of real-world measurable control properties like aerial spray percent knockdown, residual barrier spray efficacy time, and larval source recovery time, for example. The precise mathematical form of these expressions are given in the subsections below. Generally, control strengths are increasing functions of their application frequencies and approach zero as application frequencies approach zero. Numerical values for real-world control parameters appearing in control strength expressions are chosen based on expert opinion (Kevin Caillouet, personal communication) and the fitting results in Ref. [9], and are given in Table A. Figure A shows plots of ρ_A, ρ_D, and σ_D as functions of application frequency under our assumed model and control parameters. Following the results in Ref. [9], the individual control strengths $\rho_A(f_A), \rho_D(f_D)$ and $\sigma_D(f_D)$ combine

to give the following death and emergence rates in compliant and non-compliant sites:

$$
\begin{aligned}
\mu_N &= \mu_0 \frac{1}{1-\rho_A(f_A)} \\
\mu_C &= \mu_0 \frac{1-\rho_A(f_A)\rho_D(f_D)}{\Big(1-\rho_A(f_A)\Big)\Big(1-\rho_D(f_D)\Big)},
\end{aligned}
\tag{76}
$$

$$
\begin{aligned}
\Lambda_N &= \Lambda_0 \\
\Lambda_C &= \Lambda_0\Big(1-\sigma_D(f_D)\Big).
\end{aligned}
\tag{77}
$$

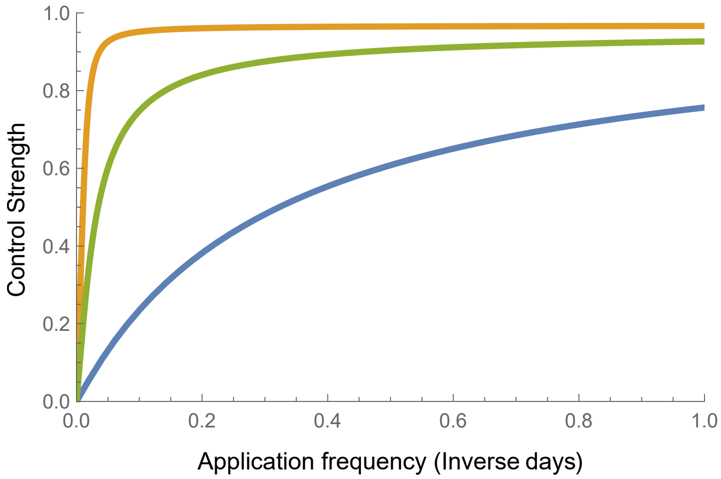

Fig. A. Control strengths as a function of application frequency in inverse days under our selected values for control and model parameters. Area-wide adulticide spray control strength ρ_A in given in blue, door-to-door residual barrier adulticide control strength ρ_D is given in orange, and door-to-door larval source reduction control strength σ_D is given in green.

6.2 Adulticide aerial spray

Area-wide spraying consists of an adulticide such as Naled, Malithion, or a pyrethoid which is dispersed throughout the neighborhood at ultra-low volumes by truck or airplane [10]. The spray remains suspended in the air for minutes to at most an hour, and kills adult mosquitoes upon contact [10]. We model adulticide aerial spray as an instantaneous fractional knockdown in mosquito population. Specifically, when adulticide aerial spray is applied to an isolated neighborhood site, the mosquito population is instantaneously reduced by the percent knockdown $\overline{\rho}$. For repeated impulses applied at frequency f_A, Ref. [9] shows the associated control strength $\rho_A(f_A)$ in Eq. (76) to be

$$
\rho_A(f_A) = \frac{\overline{\rho}}{1-(1-\overline{\rho})e^{-\mu_0 f_A^{-1}}} \frac{1-e^{-\mu_0 f_A^{-1}}}{\mu_0 f_A^{-1}}. \tag{78}
$$

6.3 Door-to-door adulticide

Residual barrier spray consists of a long-lasting adulticide such as bifenthrin or lambda-cyhalothrin, which is applied by individuals (with backpack mounted or hand sprayers, for example) to vegetation and other potential mosquito landing sites [10]. Adult mosquitoes who land on treated surfaces are quickly killed, and killing efficacy is typically retained for days to weeks as the insecticide evaporates away [10]. We model residual barrier spray as an instantaneous increase in vector death rate, and a subsequent recovery to the the natural value. Specifically, when door-to-door adulticide is applied to an isolated neighborhood site, the vector death rate is instantaneously increased by a fraction $\overline{\gamma}$, and the increase decays exponentially at rate η (meaning that $1/\eta$ is the barrier spray efficacy time). For repeated impulses applied at frequency f_D, Ref. [9] shows the associated control strength $\rho_D(f_D)$ in Eq. (76) to be

$$\rho_D(f_D) \quad = \quad 1 - f_D \int_0^{f_D^{-1}} \mathrm{d}s \left[g(s)I(s) + g(s)\frac{g(f_D^{-1})I(f_D^{-1})}{1-g(f_D^{-1})} \right], \tag{79}$$

where we define

$$g(s) \quad = \quad \exp\left[-\mu_0 s - \overline{\gamma}\frac{\mu_0}{\eta}\left(1-e^{-\eta s}\right) \right], \tag{80}$$

and

$$I(s) \quad = \quad \int_0^s \mathrm{d}s' \mu_0 \exp\left[\mu_0 s' + \overline{\gamma}\frac{\mu_0}{\eta}\left(1-e^{-\eta s'}\right) \right] \tag{81}$$

$$\quad = \quad \frac{\mu_0}{\eta}\exp\left[\overline{\gamma}\frac{\mu_0}{\eta}\right]\left(\overline{\gamma}\frac{\mu_0}{\eta}\right)^{\frac{\mu_0}{\eta}} \Gamma\left[-\frac{\mu_0}{\eta}, \overline{\gamma}\frac{\mu_0}{\eta}e^{-\eta s}, \overline{\gamma}\frac{\mu_0}{\eta} \right], \tag{82}$$

where $s > 0$ and Γ denotes the doubly incomplete gamma function:

$$\Gamma\left[a, z_0, z_1\right] \quad = \quad \int_{z_0}^{z_1} \mathrm{d}t\, t^{a-1}e^{-t}. \tag{83}$$

6.4 Door-to-door larval source reduction

Larval source reduction consists of employees identifying and eliminating receptacles for standing water typically found in residential yards (such as tires or empty buckets) which can serve as habitats for mosquito larvae. We model larval source reduction as an instantaneous decrease in vector emergence rate, and subsequent recovery to the natural value. Specifically, when door-to-door larval source reduction is applied to an isolated neighborhood site, the vector emergence rate is instantaneously decreased by a fraction $\overline{\sigma}$, and the emergence rate recovers exponentially at rate ν (meaning that $1/\nu$ is the carrying capacity recovery time). For repeated impulses applied at frequency f_D, Ref. [9] shows the associated control strength $\sigma_D(f_D)$ in Eq. (77) to be

$$\sigma_D(f_D) \quad = \quad \overline{\sigma}\frac{1-e^{-\nu f_D^{-1}}}{\nu f_D^{-1}}. \tag{84}$$

6.5 Parameter values

Parameter	Description	Value
$\overline{\rho}$	Adulticide aerial spray percent knockdown	0.20
$\overline{\gamma}$	Door-to-door adulticide fractional death rate increase	30
$1/\eta$	Door-to-door adulticide efficacy time	12 days
$\overline{\sigma}$	Larval source reduction fractional carrying capacity decrease	0.95
$1/\nu$	Larval source reduction efficacy time	20 days
c_A	Cost per aerial spray application	$24.10
c_{DN}	Cost per door-to-door application to 100 non-compliant sites	$75.52
c_{DC}	Cost per door-to-door application to 100 compliant sites	$422.90

Table A. Control strength and cost parameters used in numerical simulations. The cost per application for all controls, adulticide aerial spay percent knockdown, and larval source reduction strength and efficacy time are based on expert opinion (K. Caillouet, personal communication). The strength and efficacy for door-to-door adulticide are taken from the experimental-based model fitting in Ref. [9]

7 20% compliance optimized control for finite hopping rates

Here, we present optimized control results for finite hopping rates analogous to those given in the main text, but for dispersed and clustered 20% compliance distributions under both periodic and reflecting boundary conditions. The plots are given given in Fig. B. At 20% compliance, for the clustered distribution in Fig. Bf under either boundary condition, optimal control action calls for combined strategies for hopping rates $\omega \in (6.20\mu_0, 10.5\mu_0)$. For hopping rates below and above this interval, optimal control action calls for aerial spray only and door-to-door control only, respectively. The corresponding intervals for the dispersed 20% compliance distribution are given by $(1.30\mu_0, 1.90\mu_0)$ under periodic boundary conditions and $(1.70\mu_0, 2.30\mu_0)$ under reflecting boundary conditions. For each boundary condition and compliance configuration pictured in Fig. Bf, there exists a hopping rate interval where system is controllable under door-to-door control alone, but is more expensive to control with door-to-door control than with aerial spray. For hopping rates below this interval (excluding $\omega = 0$), the system is uncontrollable with door-to-door control alone, and door-to-door only control is optimally applied as frequently as possible (once per day). Above this interval, the system is controllable under door-to-door only and is cheaper to control than with aerial spray only. The intervals are given by $(6.20\mu_0, 8.50\mu_0)$ for the clustered distribution under either boundary condition, $(1.30\mu_0, 1.60\mu_0)$ for the dispersed distribution under periodic boundary conditions, and $(1.70\mu_0, 2.00\mu_0)$ for the dispersed distribution under reflecting boundary conditions.

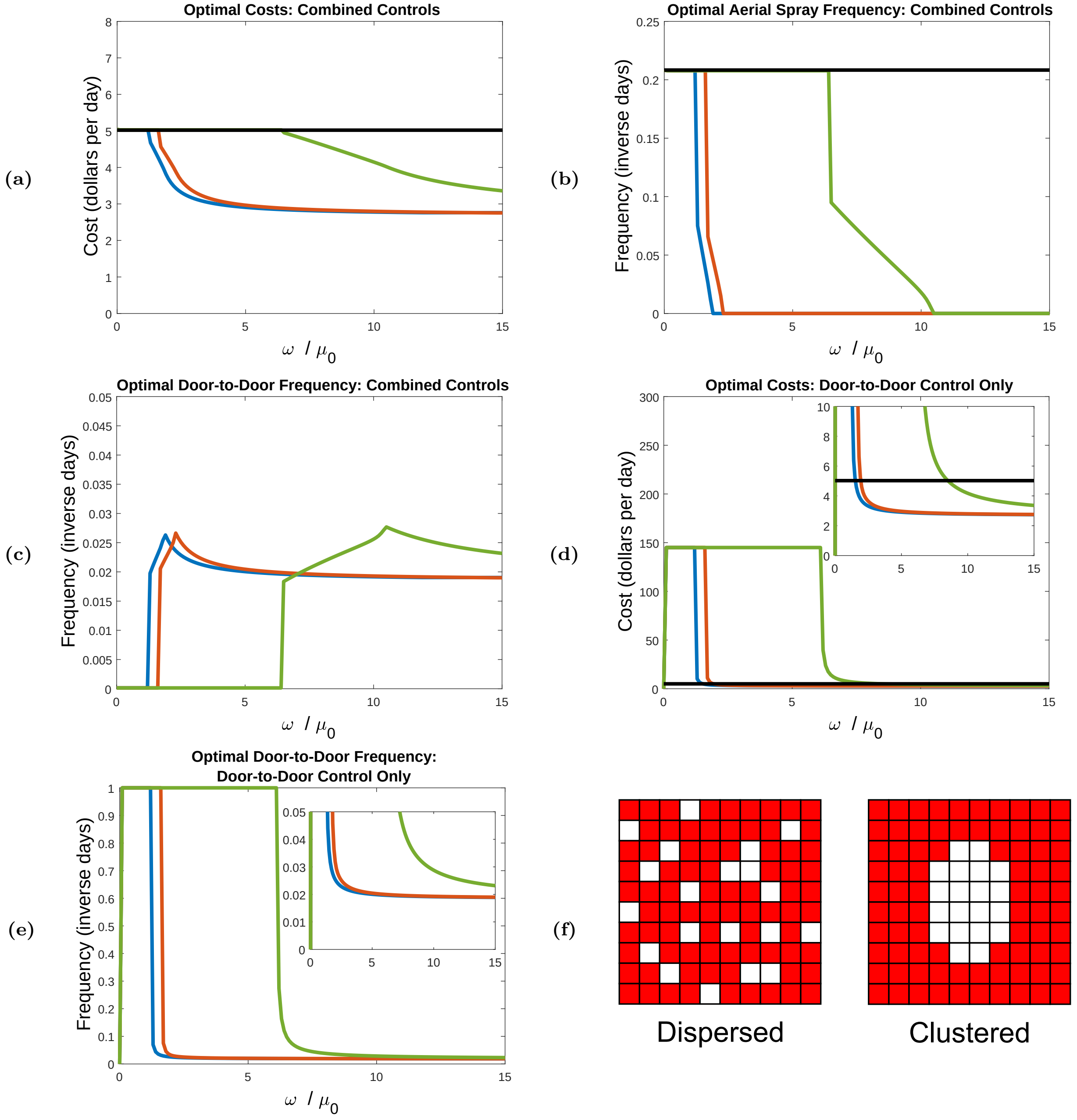

Fig. B. Optimal controls and application frequencies for the 20% compliance dispersed and clustered distributions in Fig. Bf, where white squares indicate compliant sites, and red squares indicate non-compliant sites. Blue and red curves correspond to the dispersed distribution under periodic and reflecting boundary conditions, respectively. The differences in costs and frequencies between reflecting and periodic boundary conditions is negligible for the clustered distributions, and the green curves in the figures correspond to the clustered distribution under either reflecting or periodic boundaries (these curves coincide). The black line in Figs. Ba, Bb, and Bd corresponds to aerial spray control only (e.g. used without door-to-door control). The crossover points in the zoomed-in inset in Fig. Bd are the cut-off hopping rates beyond which door-to-door control alone becomes more cost-effective than aerial spraying alone.

8 Total Epidemic Size

In Figs. C and D, we show the relationship between the total epidemic size of infectious hosts and $\mathcal{R}_0$, assuming either door-to-door only or aerial spray only control strategies. The four plots in Fig. C correspond to door-to-door only control under the 20% compliance distributions in Fig. Bf and the 60% compliance distributions in Fig. 7f of the main text, and Fig. D corresponds to aerial spray only control (where compliance is irrelevant). Boundary conditions are taken to be periodic. For each compliance configuration and control strategy, we simulate the full disease dynamics with initial conditions given by the susceptible disease-free equilibrium state plus a single infectious vector introduced into a non-compliant site. For each simulation, we choose ω equal to either $10\mu_0, 5\mu_0, \mu_0, 0.5\mu_0$, or $0.1\mu_0$, select an application frequency between 0 and 1 day^{-1}, compute $\mathcal{R}_0$, and find the cumulative total number of infectious hosts generated over the entire course of the epidemic. Results are displayed as curves depicting the total epidemic size as a function of $\mathcal{R}_0$. Each curve represents a particular hopping rate, and arrows along the curves indicate directions of increasing application frequency such that the leftmost endpoint of each curve corresponds to the maximum application frequency 1 day^{-1}. When this point lies to the left of the dotted $\mathcal{R}_0 = 1$ line, the system is considered controllable (in the sense defined in the main text) under the corresponding door-to-door only or aerial spray only strategy.

Figures C and D show that for a given set of system parameters and initial conditions, both $\mathcal{R}_0$ and total epidemic size decrease with increasing application frequency. In other words, when controlling a system using either aerial spray only or door-to-door only strategies, total epidemic size decreases monotonically with the degree to which $\mathcal{R}_0$ is reduced. For systems which are controllable, Figs. C and D show that reducing $\mathcal{R}_0$ to or below one ensures the total epidemic to be small and contained.

For all controllable combinations of hopping rate and compliance distribution considered in Fig. C, with one exception, the combined cost-optimal $\mathcal{R}_0 = 1$ controls as calculated in the main text call for either door-to-door only strategies or aerial spray only strategies. For these cases, the total size relations at $\mathcal{R}_0 = 1$ under the cost-optimal strategy are pictured in either Fig. C or Fig. D. Specifically, for systems which are controllable under door-to-door only in Fig. C, door-to-door only control is the optimal strategy, and the final size relation at $\mathcal{R}_0 = 1$ is thus given in Fig. C. For systems which are uncontrollable under door-to-door only (meaning those curves in Fig. C with leftmost endpoint to the right of the $\mathcal{R}_0 = 1$ line), the cost-optimal control is an aerial spray only strategy, and the corresponding $\mathcal{R}_{=}1$ total size relations are given in Fig. D. The exceptional case is the 20% clustered compliance distribution with hopping rate $\omega = 10\mu_0$ (the teal curve in Fig. Cd). Here, the system is controllable under door-to-door only, and the cost-optimal control calls for a combined door-to-door and aerial spray strategy. The cost optimal total size at $\mathcal{R}_0 = 1$ is found to be 11.9 hosts, which is comparable to the door-to-door and aerial spray only values of 15.0 and 16.2 hosts, respectively. We thus conclude that cost-optimal strategies for reducing $\mathcal{R}_0$ to one coincide with strategies which reduce outbreaks to small sizes. It is important to note that cost-optimal controls for reducing $\mathcal{R}_0$ to one are not not equivalent to optimal controls for reducing the total epidemic size. Specifically, combined aerial spray and door-to-door strategies which bring $\mathcal{R}_0$ to one while minimizing the total epidemic size are unlikely to coincidentally minimize the cost of control. Additionally, cost-optimal controls for reducing the total epidemic size (rather than for reducing $\mathcal{R}_0$) will be time-dependent, and finding them will require more sophisticated optimal control theory tools.

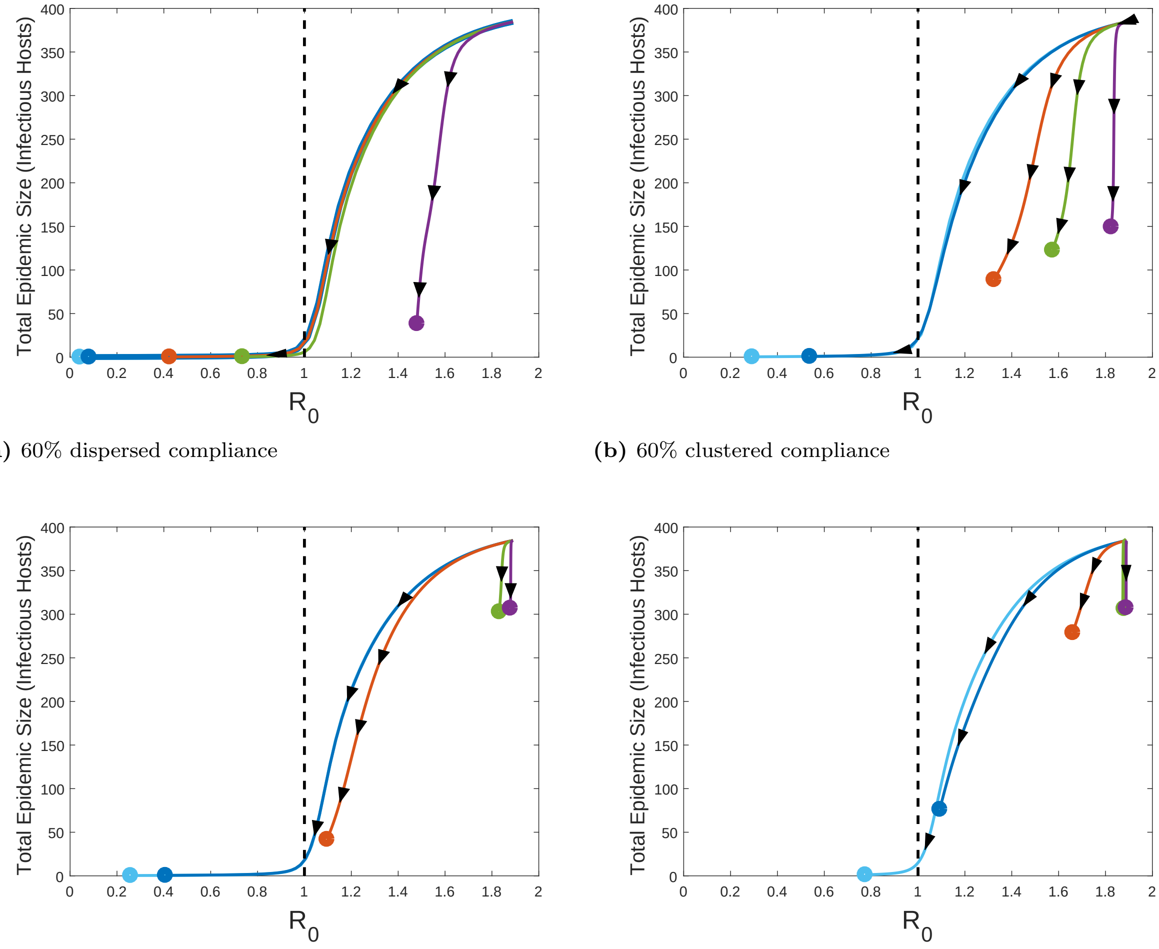

(a) 60% dispersed compliance

(b) 60% clustered compliance

(c) 20% dispersed compliance

(d) 20% clustered compliance

Fig. C. Door-to-door only control: Relation between the total epidemic size of infectious hosts and the basic reproduction number for door-to-door only control strategies. Each plot corresponds to one of the 20% compliance distributions in Fig. Bf or one of the 60% compliance distributions in Fig. 7f of the main text. Teal, blue, red, green, and purple curves correspond to the hopping rates $\omega = 10\mu_0, 5\mu_0, \mu_0, 0.5\mu_0$, and $0.1\mu_0$, respectively, and the dotted vertical line indicates $\mathcal{R}_0 = 1$. Each curve shows the total epidemic size as a function of $\mathcal{R}_0$ for a fixed initial condition and various door-to-door application frequencies between 0 and 1 $(\text{day})^{-1}$. Black arrows along curves indicate directions of increasing application frequency. In a given plot, the rightmost endpoints of all curves converge to the uncontrolled $\mathcal{R}_0$ value 2.87 (representing an application frequency of 0), and the leftmost endpoints marked with large dots represent the maximum application frequency 1 $(\text{day})^{-1}$. All figures show that the total outbreak size will be small whenever $\mathcal{R}_0 \leq 1$.

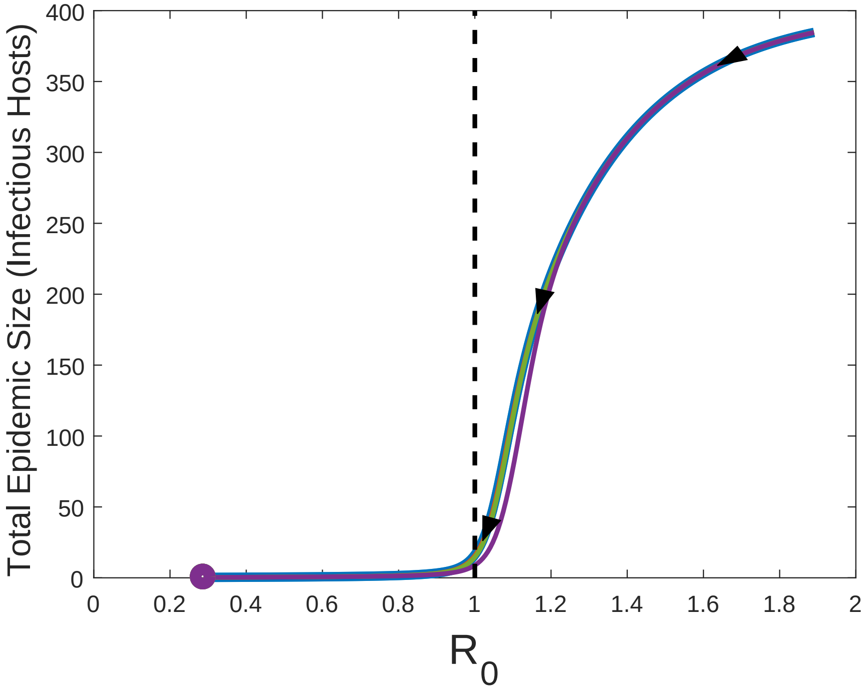

Fig. D. Aerial spray only control: Relation between the total epidemic size of infectious hosts and the basic reproduction number. Teal, blue, red, green, and purple curves correspond to the hopping rates $\omega = 10\mu_0, 5\mu_0, \mu_0, 0.5\mu_0$, and $0.1\mu_0$, respectively. Each curve indicates the total epidemic size as a function of $\mathcal{R}_0$ for a fixed initial condition and various aerial application frequencies between 0 and 1 $(\text{day})^{-1}$. Black arrows along curves indicate directions of increasing application frequency, and the dotted vertical line indicates $\mathcal{R}_0 = 1$. The rightmost endpoints of converge to the uncontrolled $\mathcal{R}_0$ value 2.87 (representing an application frequency of 0), and the leftmost endpoints marked with large dots represent the maximum application frequency 1 $(\text{day})^{-1}$. All curves nearly completely overlap one another, indicating that the total size relation is insensitive to hopping rate for aerial spray only control strategies.

References

1. van den Driessche P, Watmough J. Reproduction numbers and sub-threshold endemic equilibria for compartmental models of disease transmission. Math Biosci. 2002;180:29–48.

2. van den Driessche P, Watmough J. Further notes on the basic reproduction number. In: Brauer F, Wu J, van den Driessche P, editors. Mathematical Epidemology. Berlin Heidelberg: Springer-Verlag; 2008. p. 159–178.

3. Mohar B. Graph Laplacians. In: Beineke LW, Wilson RJ, editors. Topics in Algebraic Graph Theory. Cambridge: Cambridge University Press; 2005. p. 113–130.

4. Zhang F. Matrx Theory: Basic Results and Techniques. 2nd ed. New York Dordrecht Heidelberg London: Springer; 2011.

5. Tien JH, Shuai Z, Eisenberg MC, van den Driessche P. Disease invasion on community networks with environmental pathogen movement. J Math Biol. 2015;70:1065–1092.

6. Sakurau JJ, Napolitano J. Modern Quantum Mechanics. 2nd ed. San Francisco: Addison-Wesley; 2011.

7. Kato T. Perturbation Theory for Linear Operators. 2nd ed. Berlin Heidleberg New York: Springer-Verlag; 1995.

8. Berman A, Plemmons RJ. Non-Negative Matricies in the Mathemaitcal Sciences. 2nd ed. Philadelphia, PA: Society for Industrial and Applied Mathematics; 1994.

9. Demers J, Robertson SL, Bewick S, Fagan WF. Implicit versus explicit control strategies in models for vector-borne disease epidemiology;. BiorXiv:10.1101/753475v1 [Preprint]. 2019 [cited 2019 September 5]. Available from: https://www.biorxiv.org/content/10.1101/753475v1.

10. Faraji A, Unlu I. The eye of the tiger, the thrill of the fight: Effective larval and adult control measures against the asian tiger mosuito, *Aedes albopictus* (Diptera: Culicidae), in North America. J Med Entomol. 2016;53:1029–1047.
