# Supplementary material for: Managing disease outbreaks: The importance of vector mobility and spatially heterogeneous control
Source: PLoS Comput Biol. 2020 Aug 21;16(8):e1008136. doi: 10.1371/journal.pcbi.1008136 (PMC7480881; doi:10.1371/journal.pcbi.1008136)
Supplement: Attachment — Submitted filename: Response_To_Reviewers.docx [file pcbi.1008136.s003.docx]

To the editor and reviewers,

The authors thank the reviewers and editor for their time and thoughtful responses. In response to the general comments from the reviewers and editor, we have eliminated 14 pages of material, reduced the number of equations from 56 to 24, and removed two figures. The removed material is now included in the S1 Appendices supporting information document. To emphasize the central results and main ideas of our paper, we have added a two paragraph “Summary of main results” subsection at the beginning of the discussion section. This summary lists the key results which are explained in more detail within the bulk of the discussion section and allows the reader to quickly locate and assimilate the most important biological and practical implications of our work. These changes more sharply focus our paper on biology and practical disease management advice, rather than on technical aspects related to the underlying mathematics.

Specific major reductions are as follows. The material on the equilibrium vector population in Methods I and most of the technical derivations, definitions, and equations originally in Methods II have been translated to appendices in the supporting information. The only pieces remaining are the minimal set of definitions and expressions which are necessary for describing our problem set-up and explaining the results. Additionally, much of the material describing the relationship between variations in controlled model parameters and real-world control application frequencies which was originally in Methods III has been moved to the supporting information. Further, we have moved what was originally Figure 8 into the supporting information, as the original Figures 8 and 9 conveyed similar information. The content of the original appendices has likewise been integrated into the supporting information, except for the original Table 3 specifying model parameter descriptions and numerical values. The original Table 3 has been expanded and is now located in the Methods I section where we develop our model. Although we have made large content reductions, the biological and practical implications of our results can be described entirely in terms of the material remaining in the reformatted Methods and Results sections, and readers seeking more mathematical detail can consult the supporting information.

We have included a clean version of the revised manuscript, a marked version which highlights new additions and any changes to the portions of the original text which has not been removed (some of which includes addressing a typos and rewording some passages to accommodate the removed material), and the new supporting information document. On the following pages, we respond to the each of the reviewer’s specific comments. We appreciate being given the opportunity to improve our manuscript and we look forward to hearing further from the editor and reviewers.

J. Demers

**Responses to reviewer 1**

**Reviewer Comment:** “It is insufficient for the validity of controlling only R0 (the basic reproduction number less than unity) in their spatial model. In general, the basic reproduction number for a single-patch mathematical model is the key indicator whether outbreaks will occur or not. However, it is not clearly shown whether it would be the case for multi-patch models. For example, multi-patch models for vector-borne diseases with human movement showed disease persistence even though R0 is less than 1 in each patch (JTB 2009, The Effects of Human Movement on the Persistence of Vector- Borne Diseases). The results highlight human movement can cause the disease persistence even when R0 < 1.”

**Response:** We respectfully disagree with this assessment. In our work, we are careful to define and calculate the basic reproduction number using the next-generation matrix formalism. Under this formalism, R0 is guaranteed to reflect the local stability of the disease-free equilibrium for compartmental models, as shown by van den Driessche and Watmough (Math. Biosci. 2002, Reproduction Numbers and Sub-Threshold Endemic Equilibria for Compartmental Models of Disease Transmission). We stress that this holds true because we focus on controlling the *global* R0, rather than the *single-patch* R0 values. This is in fact shown explicitly for the model in the paper cited by reviewer 1 (JTB 2009, The Effects of Human Movement on the Persistence of Vector- Borne Diseases). Here, the disease-free equilibrium is stable when the global R0 is less than one, and unstable when greater than one – this holds true independently of the single-patch R0 values, regardless of the fact that the global R0 may be greater than one when the single-patch R0’s are less than one. In our work, we make no assumptions about the relevance of the single-patch R0’s to the global R0. Through our mathematical analysis, however, we are able to show that the single-patch compliant and non-compliant R0 values place lower and upper bounds on the possible values of the global R0 for any hopping rate, control strategy, and compliance distribution. We do not claim that the global R0 can always be reduced to one by reducing some or all of the single-patch R0’s to any particular value, and one of our central results shows that the ability to do so with door-to-door control in compliant sites only is highly dependent on the compliance level, degree of clustering, and hopping rate. To emphasize this central result in the revised manuscript, we summarize it briefly in the new “Summary of main results” subsection, specifically on lines 695-706.

We also remark that endemic disease persistence will never be an issue in our model. We include no host births or deaths, and we assume that all infected hosts eventually recover and do not lose immunity, so there exists no mechanism by which the susceptible host class can be repopulated and allow the disease to persist in the host and vector populations. A large outbreak may well burn through most of the host population, but it will never persist indefinitely. For our model, then, the global R0 is simply a useful quantity which determines whether or not an outbreak can occur in an otherwise disease-free system and serves as a proxy measure for the expected initial rate of disease growth.

**Reviewer Comment:** “Authors should provide extensive analysis on the final size relation (the total number of infected and the basic reproduction number) and furthermore, the effects of vector mobility on the final size relation should be discussed as well.”

**Response:** We agree that final size relation would be an interesting quantity to study, but we feel that this falls outside of the scope of our work. Our focus is on preventative control in a susceptible disease-free area, which is why we concentrate on reducing R0. The actual final size relation is not especially relevant to our control goal – once an epidemic has occurred and created large numbers of infected host and vectors, preventative control protocols suggested by R0 reduction are unlikely to be effective strategies, and optimally managing the outbreak is an entirely different control problem which requires use of the full disease dynamics and optimal control theory techniques. One of the major points of our paper is to show that optimal preventative control through R0 reduction can be formulated as simple linear constrained optimization problem which is more accessible to those without an advanced mathematical background, as opposed to the fully dynamic optimal control theory problem for reducing the final outbreak size. To emphasize this point in the revised manuscript, we summarize it briefly in the new “Summary of main results” subsection, specifically on lines 706-711.

We also remark that, as reviewer 1 indicates, a thorough and meaningful final size analysis would be extensive in nature. Not only would we need to consider many hopping rates, compliance levels, compliance distributions, and control strengths (as we do in our current work), but also many spatial distributions for the initial infection. The particular features of the R0 reduction problem allow for analytical tractability in a select number of limiting cases, and the insights afforded by this tractability allow us to make inferences into more general realistic cases, thus allowing us to form a useful and complete picture of solutions to the R0 reduction problem without resorting to large numbers of numerical simulations. The final size relation, however, is analytically intractable even in simplified limiting cases, so a very large number of ODE simulations would be required to obtain a complete picture. In short, an extensive final size relation analysis is not intimately related to the topic of our paper and is worthy of a separate paper in its own right, and given the length of our manuscript as it stands, all of this interesting material would need to be relegated to supplementary sections.

**Reviewer Comment:** “Therefore, the authors should carefully validate if it is sufficient to control only the basic reproduction number.”

**Response:** As outlined in the above responses, R0 reduction is sufficient for the particular goal of preventative control in a disease-free area. R0 reduction may not be sufficient for other control goals, such as reducing the final outbreak size, but alternative control goals fall outside the intended scope of our work.

**Responses to reviewer 2**

**Reviewer Comment:** “1. In Methods II, the authors walk carefully through the process of how the basic reproductive number was obtained. This section was perhaps the most dense of the methods section and it read as material more appropriate for a theoretical biology or applied mathematics journal. Would it be possible to break this section down into key points and leave details to the appendices? If this approach was taken, the authors could focus on the R0 expressions obtained (such as those in equation 23) and explain the importance of each. The authors could then follow up with the hopping scenario-specific discussions of R0.”

**Response:** We have greatly reduced the length and mathematical detail of all Methods sections, most prominently in Methods II. The removed material is now in the S1 Appendices supporting information document. The only material remaining in Methods II is the statement and meaning of our R0 eigenvalue problem, the various R0 expressions, and the minimal elements needed to coherently describe and discuss the perturbation theory results.

**Reviewer Comment:** “2. Related to the hopping scenarios: It would be interesting to get the authors' perspectives on how each of these scenarios relates to different mosquitoes and mosquito borne disease. It seems like finitely slow hopping is the only realistic scenario of the three. These scenarios are mathematically interesting, but are they biologically relevant?”

**Response:** We attempted to address this topic in the discussion section on lines 958-971 of the original manuscript. In the revised manuscript, this passage has been slightly modified and moved to a more relevant location: lines 197-210, directly after the introduction of the mosquito motion model and the definition of the hopping rate. Here, we give estimated hopping rates for *Aedes albopictus* and *Aedes aegypti* based on previous modeling studies, and an issue is immediately apparent - the two estimates for *Aedes aegypti* vary by over two orders of magnitude. We suspect that this inconsistency arises from a lack of experiments necessary for determining hopping rates and diffusion constants. Such an experiment would need to consist of a mark capture release study where a very large number of mosquitoes are released at a single point in space and time in a fairly homogeneous area, and the mosquito positions would need to presented as a function of time. In preparing the original manuscript, we searched for papers describing such experiments from which we could estimate our own diffusion constants and hopping rates for various species, but were unable to find any relevant studies. Most experiments seem to capture but not release mosquitoes, and their inability to continue moving over their natural lifetimes would lead to potentially severe underestimates of the hopping rate. Further, the studies we found did not indicate a maximum distance beyond which no mosquitoes were able to be trapped, so the actual mean square displacement for a species over its natural lifetime could not be easily extrapolated. Rather than speculate with a lack of experimental data, we thought it best to give numbers from the few theoretical studies we could find which actually associate a diffusion constant to a particular species, and give a brief description of the type of experiment needed to obtain accurate hopping rate estimates.

Regarding the relevance of the fast hopping and no hopping scenarios, these are indeed idealized limits which are likely biologically unrealistic. However, they are analytically tractable limits, and they place bounds on what can be expected in more realistic scenarios which are analytically intractable. These idealized limits are important in that they are part of the basis from which we make inferences to form a complete picture of solutions to the R0 reduction problem for more realistic hopping rates, and to understand the behavior of the optimized control results. The no-hopping scenario is especially important - the more realistic finitely slow hopping perturbation formalism cannot be defined without the no-hopping limit (finitely slow hopping is a perturbation away from the no-hopping scenario). We have added a sentence emphasizing this point on lines 269-272 of the revised manuscript.

**Reviewer Comment:** “3. I think interpretation of the results would benefit from a table of parameter descriptions in the main text. The table could include only those parameters that are important to reading and understanding figures.”

**Response:** We have added a table (Table 2 in the revised manuscript) to address this comment at the beginning of the Results I section of the revised manuscript. Also, we have expanded and moved the model parameter summary table (Table 1 in the revised manuscript) into the model development section (this table originally appeared in the appendices).

**Reviewer Comment:** “4. It would also be worthwhile to have more clear reasoning behind the choice of metrics in figures. For instance, why do the authors present the metric on the vertical axis in Fig 6? What is the value of looking at omega/mu_0 in Figs 8-9? Are there other metrics that would perhaps be easier to interpret biologically for these results?”

**Response:** We have added passages throughout the revised manuscript to address this comment and have summarized the meaning of these quantities in the new Table 2. The passages can be found in the revised manuscript on lines 475-476, 503-507, 511-515, 527-529, and 619-624. In short, some metrics are chosen because they most transparently represent a notion of control strength or effort, and some metrics are chosen so that the results presented are as independent as possible from the particular numerical values chosen for our simulations, and instead represent features universally inherent to the structure of our model. Regarding the specific cases mentioned by reviewer 2, in Fig. 5 (which was Fig. 6 in the original manuscript), we scale out kappa because delta R0 depends only linearly on kappa. By scaling out kappa, Fig. 5 applies regardless of the values used for model parameters and is in this sense maximally informative. In Fig. 7 (which was originally Fig. 9, the original Fig. 8 has been moved to the supporting information), we use omega / mu_0 as opposed to just omega because mu_0 sets a natural time scale for the mosquito population dynamics from which one can quantify “fast” and “slow” hopping, and omega / mu_0 does not depend on the units chosen for time. Hopping is fast for omega / mu_0 >> 1, and slow for omega / mu_0 << 1. Omega / mu_0 is represents the average number of hops a mosquito makes in a lifetime, and is more intuitive to interpret than omega alone, whose magnitude is unit dependent.

**Reviewer Comment:** “5. What are they key results here? There were a number of interesting figures presented in the results; however it was difficult to parse the major points from the work as it is presented.”

**Response:** We have added a “Summary of main results” subsection at the beginning of the discussion section (lines 683-711) which highlights the key results of our work. A reader should be able to quickly find and jump to this section if desired.

**Reviewer Comment:** “6. I think this work is very interesting and useful overall, but I worry that there is too much information presented here, which distracts from the intended message of the paper.”

**Response:** We have attempted to address this comment by reducing the amount of technical material in all methods sections, reducing the number of figures, and adding the “Summary of main results” subsection to the beginning of the discussion section.
