# Supplementary material for: Managing disease outbreaks: The importance of vector mobility and spatially heterogeneous control
Source: PLoS Comput Biol. 2020 Aug 21;16(8):e1008136. doi: 10.1371/journal.pcbi.1008136 (PMC7480881; doi:10.1371/journal.pcbi.1008136)
Supplement: Attachment — Submitted filename: Second_Response_To_Reviewers_7-5-2020.docx [file pcbi.1008136.s004.docx]

7/5/2020

To the editor and reviewers,

We again thank the reviewers and editor for their time and consideration. All changes to the previous revision of the main text and supporting information are highlighted in the marked-up versions. In response to reviewer 1’s comment, we have conducted numerical experiments using the full disease dynamics to find the total epidemic size of infectious hosts. To show the relation between control of the total epidemic size and control of the basic reproduction number, we plot the total size vs. R0 for all control application frequencies between the upper and lower bounds 0 and 1, assuming fixed choices for compliance structure, hopping rate, and initial conditions. To avoid both lengthening our manuscript and distracting from our paper’s primary focus on preventative R0 control, we have included the 3 pages of new text and figures as an additional appendix section on pages 25-28 of the S1 Appendices Supporting Information document. We now mention this appendix on lines 370-376 of the main text, and we have updated the supporting information legend on lines 1018-1019.

The new material shows that when controlling any particular neighborhood system for a given initial condition, both R_0_ and total epidemic size decrease as control application frequency increases, and that the total epidemic size decreases monotonically as R_0_ decreases. Additionally, we find that reducing R_0_ to or below 1 is sufficient for containing the total outbreak to a small size. From these results, we conclude that control strategies designed for cost-optimal R_0_ reduction are also effective strategies for reducing the total outbreak size, and that R_0_ = 1 is also a reasonable control goal for containing active outbreaks.

We do not conclude, as reviewer 1 suggests, that “optimizing R_0_ is equally as good as optimizing the total epidemic.” We did not make this claim in the original manuscript, and we do not make it now. It is almost certain that this claim is false, due in part to the fact that the total epidemic size depends on the full non-linear disease dynamics, while R_0_ depends on the linearized disease dynamics. It is also unclear what the phrase “equally as good as optimizing the total epidemic” precisely means. If the phrase is interpreted to mean that controls which cost-optimally reduce R_0_ to 1 are equivalent to controls which epidemic size-optimally reduce R_0_ to 1, it would generally be untrue, as monetary cost and total size are entirely different control objectives. If the phrase is interpreted to mean that controls for cost-optimally reducing R_0_ to 1 are equivalent to controls for cost-optimally reducing the total epidemic size, it would generally be untrue, as cost-optimal total epidemic reducing strategies will be inherently time-dependent, while R_0_ and its controls are inherently time-independent. Our work shows that cost-optimal strategies for reducing R_0_ are also effective at reducing the total epidemic size but does not address optimal strategies for total epidemic reduction. To understand the relationship between optimal R_0_ reduction and optimal total size reduction strategies, one would need to conduct an involved optimal control theory study of the full disease dynamics, and this is outside the scope of our work.

In addition to changes the changes in response to reviewer 1’s comment, we have also found and fixed several typographic errors in the main manuscript, specifically on P. 17 line 401, P. 19 line 454, in table 2 on P. 20, and P. 36 line 700. Further, we have provided more complete funding information for one of our co-authors F. Agusto. We appreciate the opportunity to further improve our manuscript and look forward to further feedback.

J. Demers
